# Introducing Memory in Coarse-Grained Molecular Simulations

**DOI:** 10.1021/acs.jpcb.1c01120

**Published:** 2021-05-13

**Authors:** Viktor Klippenstein, Madhusmita Tripathy, Gerhard Jung, Friederike Schmid, Nico F. A. van der Vegt

**Affiliations:** † Eduard-Zintl-Institut für Anorganische und Physikalische Chemie, Technische Universität Darmstadt, 64287 Darmstadt, Germany; ‡ Institut für Theoretische Physik, Universität Innsbruck, Technikerstraße 21 A, A-6020 Innsbruck, Austria; § Institut für Physik, Johannes Gutenberg-Universität Mainz, Staudingerweg 9, 55128 Mainz, Germany

## Abstract

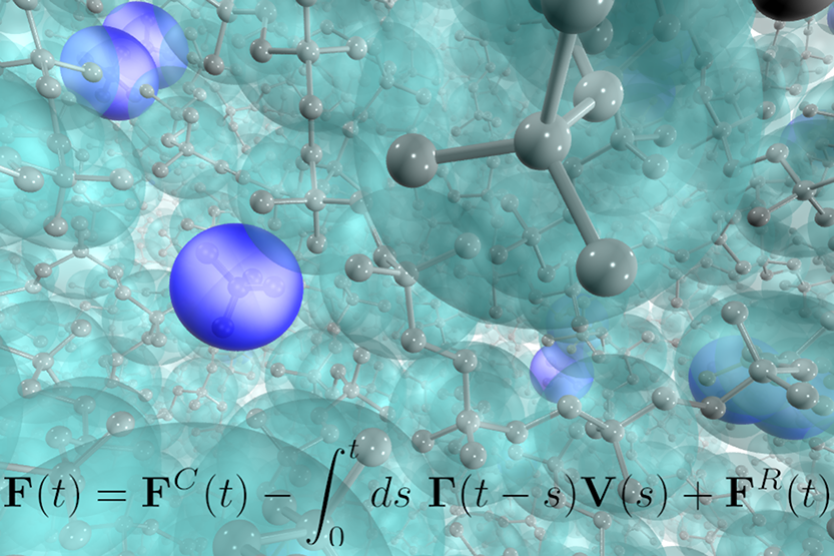

Preserving the correct dynamics at the coarse-grained (CG) level is a pressing problem in the development of systematic CG models in soft matter simulation. Starting from the seminal idea of simple time-scale mapping, there have been many efforts over the years toward establishing a meticulous connection between the CG and fine-grained (FG) dynamics based on fundamental statistical mechanics approaches. One of the most successful attempts in this context has been the development of CG models based on the Mori–Zwanzig (MZ) theory, where the resulting equation of motion has the form of a generalized Langevin equation (GLE) and closely preserves the underlying FG dynamics. In this Review, we describe some of the recent studies in this regard. We focus on the construction and simulation of dynamically consistent systematic CG models based on the GLE, both in the simple Markovian limit and the non-Markovian case. Some recent studies of physical effects of memory are also discussed. The Review is aimed at summarizing recent developments in the field while highlighting the major challenges and possible future directions.

## 1 Introduction

The development of methods for dynamically consistent systematic coarse-grained simulations is a relatively new and promising research area in the field of soft matter simulations. In this Review, we discuss the current state of affairs of introducing memory effects in coarse-grained molecular simulations. We particularly focus on recent methodological advances, highlighting the underlying challenges and capabilities. For alternative approaches in the field of dynamic coarse-graining and systematic coarse-graining methods based on structural and thermodynamic properties, we refer the reader to other recent reviews.^1−5^

The 1998 twin papers by Tschöp et al.^6,7^ have been seminal in the field of systematic coarse-graining of soft matter systems. They paved a new route for linking chemistry and properties of polymers based on ideas to map between a fine-grained (FG: high resolution) and a coarse-grained (CG: low resolution) configuration space in, both, forward and backward directions. Regarding the dynamics of the CG system, they made two important observations. First, they showed that structural quantities equilibrate faster and more efficiently in CG models, which is good news from a sampling point of view. Second, in order to recover quantitatively reliable information on the dynamics of the system as well, they introduced the novel concept of time-scale mapping: They proposed to identify the (reduced) time scale in the asymptotic long-time regime of the CG molecular dynamics (MD) simulation with the corresponding experimental time scale by comparing the predicted melt viscosity (within the Rouse model) with its experimental counterpart.^6,8^ In later approaches, monomer mean-square displacements of the FG and CG models were used to define a so-called time mapping (or speed-up) factor, effectively accounting for the lost friction of the fast atomistic degrees of freedom (DoF) in the CG model.^8−11^

Applying this *a posteriori* time mapping procedure to CG MD simulation trajectories led to several successful quantitative predictions of dynamical properties on time and length scales, which went far beyond those that could be addressed with detailed atomistic simulations. These include dynamic chain scattering functions,^9^ self-diffusion coefficients, and viscoelastic properties of unentangled and entangled, high-molecular-weight, polymer melts.^12^ Furthermore, the diffusive dynamics of small penetrant molecules in a polymer matrix (ethylbenzene in polystyrene) could be described with CG models and time mapping procedures in quantitative agreement with experiments, achieving transferability over a wide range of temperatures.^13,14^ This, heuristic, time mapping technique was the first to successfully link chemistry and dynamic properties of polymers used in daily life. However, the applicability of the approach was mostly limited to homogeneous single-component systems. In the case of small penetrant diffusion in a polymer matrix, even though the temperature dependence of the penetrant diffusion coefficient was in agreement with experiments, the scaling factor differed for the two components (polymer and penetrant) within the same system and depended on the composition of the binary system.^15^

The scale (or speed-up) factors, in general, depend on the simulation state point and system properties such as polymer tacticity, solvent volume fraction, etc. Several studies have attempted to predict this speed-up factor in simulations based on relative entropy, interactions, and mechanical considerations.^16−18^ While this speed-up factor allows one to quantify the dynamics at the CG level in agreement with the FG counterpart, its choice is rather empirical. Moreover, it relies on the existence of a single CG time scale corresponding to the long-time diffusive limit. However, in multicomponent systems where the overall dynamics of a system is governed by relaxation mechanisms on distinct time scales, coarse-graining affects the various energy barriers differently, thereby accelerating the dynamics of the various components to different extents. In realistic chemical systems with a moderate degree of coarse-graining, such effects are expected to be more pronounced, and therefore, the use of a simple time-scale mapping approach is severely limited.

One way of preserving the real FG dynamics in a CG system is to apply the fundamental statistical approach based on the generalized Langevin equation (GLE), where the friction resulting from the lost DoF upon coarse-graining is explicitly taken into account. Over the past two decades, such an approach has been formalized based on the Mori–Zwanzig (MZ) theory,^19−22^ which can, in fact, be viewed as one of the first rigorous theories of systematic coarse-graining. Starting from an underlying microscopic system with Hamiltonian dynamics, the MZ formalism uses projection operators to derive an exact equation of motion (EoM) for a reduced set of relevant variables at the CG level. The resulting EoM has the form of a GLE, with frictional and random forces coupled through the fluctuation–dissipation theorem (FDT). The GLE is non-Markovian, as the instantaneous force depends on the entire dynamical history of the system, unlike the Hamiltonian EoM. However, depending on the nature of the system of interest, this “memory” can sometimes be short-lived, in which case it can be replaced by an instantaneous friction term. The GLE can then be approximated by a simpler stochastic equation: the Langevin equation (LE). While analyzing the non-Markovian GLE in simulation is nontrivial and computationally demanding, several studies have attempted to employ this approach to investigate the dynamical properties of various chemical systems. In this Review, we will highlight some of the recent works along this line.

The aim of this Review is to summarize the recent methodological developments in the field of dynamically consistent systematic coarse-graining. We particularly focus on studies which employ GLEs to analyze and/or simulate physicochemical systems based on the underlying FG dynamics. A concise, but not exhaustive, list of studies are briefly discussed to motivate the fundamental background and methodological progress. For a more general discussion on consistency of dynamics in CG simulations, readers are referred to another recent review.^5^

The present Review is organized as follows. The GLE as derived from the MZ formalism is briefly discussed in section 2. Section 3 describes selected studies that employ a Markovian approximation to the GLE. While highlighting the usefulness of the Markovian assumption, these studies also demonstrate the need to explicitly include memory effects depending on the nature of the underlying FG system. Section 4 discusses various possible ways to extract the memory kernel from FG trajectories with special focus on single diffusing particles. Strategies to go beyond single-particle systems and use GLE-based modeling in coarse-graining and multiscale modeling are reviewed in section 5. A crucial issue in such simulations is the availability of efficient GLE integrators. Different approaches have been proposed, some based on straightforward integration and some based on techniques that introduce auxiliary variables to map the GLE on a system of coupled Markovian Langevin equations in an extended space. These are discussed in section 6. Section 7 highlights selected recent studies of systems where memory effects have a qualitative impact on the dynamical behavior. We conclude in section 8 with a discussion on open questions and possible future directions.

## 2 Mori–Zwanzig Formalism

The Langevin equation (LE), introduced by Paul Langevin in 1908,^23^ is a prototypical example of a CG EoM. It is used to model the dynamics of a heavy Brownian particle dispersed in a fluid and describes it solely via a dynamical equation for the momentum of the Brownian particle itself, while its interactions with the fluid particles are modeled implicitly by frictional dissipation and impacts. For a given viscosity of the fluid and size of the Brownian particle, dynamical properties can be derived from the LE. The formal connection between the atomistic description of Brownian dynamics based on the Hamiltonian equation with all DoFs and a CG description of the form of a LE was established by Mori^19^ and Zwanzig^20^ based on a projection operator formalism.^22^ In this section, we briefly summarize the main ideas behind the Mori–Zwanzig (MZ) theory as discussed in ref (22) and recent extensions in the context of dynamic coarse-graining.

The projection operator formalism is based on the idea that any dynamical variable for a given Hamiltonian system can be described as a vector in a Hilbert space, consisting of a vector space spanned by a set of orthonormal basis functions and an inner product. The choice of the inner product is crucial for a consistent coarse-graining procedure. In equilibrium, the most common choice is the phase space integral
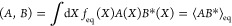
1for two arbitrary observables *A*(*X*) and *B*(*X*), phase space points *X*, and equilibrium probability distribution *f*_eq_. The inner product, (*A*, 1), thus corresponds to the usual phase space average.

In general, not all dynamical variables are of interest. For example, in coarse-graining, the central idea is to average over the fast microscopic processes and just keep a small number of slow effective variables that can represent a system on larger length and time scales. Having defined an inner product in the microscopic system now allows us to formally select some variables to be relevant (i.e., slow representatives) and others to be irrelevant via the introduction of a projection operator. Based on eq 1, a projection operator, , can be defined, which projects any dynamical variable *B* onto the subspace of relevant variables {*A*_*j*_}, as
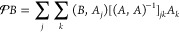
2Here, (*A*, *A*) denotes the *n* × *n* matrix of inner products (*A*_*i*_, *A*_*j*_), where *n* is the dimensionality of the relevant subspace. In the following, we will restrict ourselves to the one-dimensional case, which can easily be generalized to *n* dimensions.
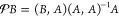
3With these definitions and starting from the Liouville equation
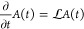
4after some mathematically exact reordering which is described in detail in ref (22), a CG EoM for *A*(*t*) can be derived as
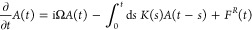
5which has the form of a generalized Langevin equation (GLE). Here we have introduced the frequency matrix
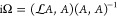
6and the “noise”
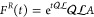
7where  is the projector on the irrelevant dynamical variables. The extended time-evolution operator, , is often referred to as “orthogonal”, “projected”, or Q-dynamics. Finally, the memory kernel is formally given by
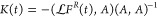
8

Equation 5 is an exact reformulation of the original Liouville equation. Being in the form of a GLE, the interpretation of *F*^*R*^(*t*) as a random process allows one to model the irrelevant variables of the original problem by a stochastic process with equivalent statistical properties. To illustrate the meaning of the separate terms in eq 5, we can assume the simplest case, in which the relevant variable is given by the momentum of a single particle *A*(*t*) = *p*(*t*). We can then write the frequency matrix Ω as
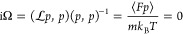
9where  is the total force on the tagged particle. Here, Ω vanishes due to the fact that the dynamics are time-translationally invariant and the Liouville operator is anti-Hermitian. (If the microscopic dynamics is diffusive and not Hamiltonian, a similar formalism can be applied. In this case, the frequency matrix Ω might not vanish.) The scalar memory function, in this case, is given as
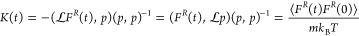
10where we have exploited  and Equation 10 relates the random force *F*^*R*^(*t*) with the memory kernel *K*(*t*) and is usually referred to as the second fluctuation–dissipation theorem (FDT). It should be noted that the derivation of the FDT only requires the assumption of an anti-Hermitian Liouville operator  and the definition of an inner product. The second FDT should thus be seen as a mathematical identity, which is valid independent of the specific choice of the inner product and which can even be extended to nonstationary systems.^24^ Having identified the different contributions to the GLE, we can rewrite the full EoM for the single Brownian particle as
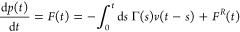
11with Γ(*t*) = *mK*(*t*).

If *A*(*t*) stands for a set of momenta of different particles rather than the momentum of a single tagged particle in one dimension, the vector *ΩA*(*t*) in eq 5 represents linearized interaction forces between the particles. Importantly, since the MZ formalism is a purely linear theory, any nonlinear contributions to the associated potential of mean force (PMF) or any nonlinear friction terms will be absorbed in the distribution of the random forces and a renormalized memory kernel.

This structure is difficult to reconcile with standard philosophies of coarse-graining, where a clear distinction is typically made between external driving forces, conservative interactions that determine the stationary distribution of the variables at thermodynamic equilibrium (the Boltzmann distribution), and dissipative forces that determine the dynamics and the entropy production in nonequilibrium.^25,26^ Making such distinctions helps to devise coarse-grained models that are thermodynamically consistent by construction, and are thus clearly desirable.

To overcome these shortcomings of the MZ formalism, modified projection operator formalisms have therefore been proposed,^27,28^ which allow conservative and dissipative forces to be separated. Kinjo and Hyodo derived the equation of motion (EoM) for CG clusters of microscopic particles. A monatomic fluid served as the microscopic system, while clusters of several atoms formed the CG particles, with centers at the respective center of masses (CoMs). The resulting CG EoM has the form of a GLE
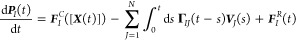
12where [***X***, ***P***] defines the 6*N*-dimensional phase space of CG particles. The first term on the rhs represents the conservative force on the CG particle *I*, which now, indeed, corresponds to the gradient of the PMF. The second term represents the friction force (dissipation) due to the removed DoFs and involves the integral of the product of the memory kernel matrix, **Γ**_*IJ*_, with the velocities ***V***_*J*_(*t*) = *M*_*J*_^–1^***P***_*J*_(*t*) of all other particles of mass *M_J_*. In general, **Γ**_*IJ*_ may be different for all pairs *I*, *J* and depend on their state (i.e., on the relative distance between particles *I* and *J*). The third term represents the random force, which is related to the friction term via the FDT
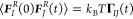
13In structural coarse-graining, multibody contributions to the PMF are often neglected and the conservative forces are pairwise decomposed, ***F***_*I*_^*C*^ ≈ ∑_*J*≠*I*_ ***F***_*IJ*_^*C*^. If one additionally neglects many-body correlations in the friction forces, eq 12 can be reformulated as^29^
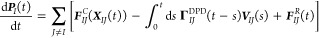
14with relative positions ***X***_*IJ*_(*t*) = ***X***_*I*_(*t*) – ***X***_*J*_(*t*) and velocities ***V***_*IJ*_(*t*) = ***V***_*I*_(*t*) – ***V***_*J*_(*t*) of particles *I* and *J*. This pairwise GLE corresponds to a non-Markovian formulation of the EoM of dissipative particle dynamics (DPD).^30^

All generalized Langevin equations presented in this section are clearly non-Markovian, but they can be reduced to Markovian variants under specific assumptions (see section 3 for details). In the case of a freely diffusing Brownian particle, the Markovian variant of the GLE (eq 11) is the standard LE
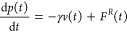
15where γ =  d*t* Γ(*t*) is the friction coefficient. The random force, *F*^*R*^, now describes uncorrelated white noise and is related to the friction coefficient via the usual FDT
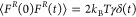
16In a similar way, the Markovian version of the pairwise GLE as derived in eq 14 can be reduced to the DPD EoM
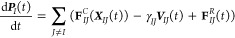
17

Since they are based on an underlying systematic coarse-graining procedure, these EoMs are thus suitable starting points for the parametrization of molecular CG models in simulations. Examples will be discussed in the following section.

## 3 The Markovian Assumption

While the evaluation of the memory kernel is a central step when constructing dynamically consistent coarse-grained models based on the GLE (eq 12), its implementation in CG simulations is technically nontrivial and computationally expensive. Therefore, Markovian approximations to the GLE have been widely used in simulations.^27,31−37^ The approach assumes the fluctuating forces to be delta-correlated in time, and not temporally correlated as in the non-Markovian case (which similarly holds for the memory kernel). The resulting EoM has the structure of a DPD equation, as defined in eq 17, and can be implemented in a relatively straightforward manner. This assumption, however, is valid only in the case where the time scales of the fast and slow variables in the system are completely separated: The time scale of the random force fluctuation must be sufficiently fast compared to the time scale of the CG bead motion. Intuitively, such an approximation should hold for high degrees of coarse-graining or systems at low density, where the atomic collisions happen on a much smaller time scale than the change in momentum of the CG beads. Whether or not this is the case can be inferred in simulations from the decay of the force and velocity auto-correlation functions (FACF and VACF): The time scales are well-separated if the former decays much faster than the latter. In contrast, in chemically specific molecular CG models with low to medium degrees of coarse-graining, the time scales of the slow and fast dynamics (the P- and Q-DoF) are not fully separated and, thus, the Markovian assumption breaks down.^35−37^ Nonetheless, the Markovian DPD has been extensively used in molecular CG models. Some examples are briefly discussed in this section.

The GLE, as derived following the MZ formalism, takes into account the projected dynamics of the underlying FG system, which is different from the real FG dynamics that one observes in a molecular dynamics (MD) simulation. In such a case, one workaround is the so-called Q-approximation, where the projected (or Q-) dynamics is approximated by the real dynamics; i.e., one assumes for the orthogonal time-evolution operator .^28,32,37^ This implies  d*t*⟨*F*^*R*^(*t*)*F*^*R*^(0)⟩ ≈  d*t*⟨*F*(*t*)*F*(0)⟩ on intermediate time scales τ. While this approach allows for an easier implementation of the CG EoM, it also leads to the well-known “plateau problem”, where the friction for finite mass CG particles, as determined from Green–Kubo integrals of the FACF, vanishes on long time scales rather than converging to a finite plateau.^38−40^ The existence of a plateau is guaranteed in the infinite mass limit, where the correlation function of the random forces in a GLE equals the correlation function of the total forces.^41^ In this limit, the large inertia of the heavy particle ensures a good separation of the time scales of the slow and fast DoFs. In this line, Sanghi et al. used the GLE to characterize memory effects in fullerene nanoparticle dynamics and investigated the scaling of the memory kernel with the nanoparticle mass, shape, and size. They observed that the FACF and the random force ACF are indeed comparable in the large nanoparticle mass limit.^42^ Nonetheless, for finite mass CG models, an intermediate plateau can be found in several cases, and the plateau values can then be taken to determine the friction coefficient.^36,37^

To circumvent the issue of time-scale separation, Hijón et al.^31^ proposed a scheme in which, by appropriately constraining the MD trajectory of the FG system, the CG dynamics was made exactly Markovian and the resulting Green–Kubo integrals were shown not to suffer from the plateau problem. The theoretical background was developed following the MZ formalism, and a star polymer melt was considered as a specific example. The modified dynamics was obtained by constraining the relevant variable, i.e., the CoM of the polymers to their respective positions in a set of configurations and carrying out short independent MD runs from each configuration. The resulting time averaged FACF and its integral (friction), calculated using the constrained MD trajectories, were found to exhibit well-defined plateaus as opposed to those calculated using unconstrained trajectories. Also, the radial distribution function (RDF) and VACF, calculated in the CG simulation, were found to be comparable to their FG counterparts.^31^

Trément et al.^34^ used the Markovian DPD approach to coarse-grain *n*-pentane and *n*-decane molecules as single DPD beads with a degree of coarse-graining (number of carbon atoms per CG bead: λ) = 5 and 10, respectively. The conservative force was calculated in constrained MD simulations as the PMF, and the normal and transverse pair frictions were calculated following Hijón et al.^31^ The random forces were calculated from the FDT as a linear combination of Wiener processes.^43^ As expected, the conservative interaction was found to be softer, while the decay of friction became slower with increasing λ. The ratio of the transverse to radial friction also increased, highlighting the role of molecular anisotropy. The models could well reproduce the RDF, the diffusion coefficient, and the viscosity of the underlying MD systems of *n*-pentane at 293 K and *n*-decane at 393 K. However, the results of the low-temperature *n*-decane DPD simulation were less convincing, owing to the anisotropic shape of the molecules and the fact that the time scales were not well separated. To check the possible transferability of the DPD force field, the authors modeled *n*-decane as a dimer of two *n*-pentane blobs, and interestingly, it could reproduce the low-temperature MD results quite well.

Lei, Karniadakis, and co-workers^32^ employed the GLE EoM as derived by Kinjo and Hyodo^28^ to study the behavior of mesoscopic clusters of Lennard-Jones (LJ) particles, constrained within a constant radius of gyration (*R*_*g*_). Under the Markovian assumption, they investigated the performance of three distinct CG models: (1) using only conservative forces, (2) using a Langevin thermostat, and (3) using a MZ DPD thermostat. The first model could only capture the FG structural properties, such as the RDF and the pressure, but not the dynamical properties, such as the diffusion coefficient and the viscosity. Furthermore, the resulting dynamical quantities could not even be matched with the corresponding FG results by simple time-scale mapping approaches.^12,15^ In the Langevin dynamics, the friction coefficient was calculated using the auto-correlation function (ACF) of the fluctuating forces, and the random forces on CG particles were assumed to be independent. The resulting diffusion coefficient was found to be 4 times smaller than that of the underlying FG system, which was attributed to the missing contribution of the configuration dependence of the frictional and random forces. In the MZ-DPD model, the random force was considered to be pairwise additive. For each pair, the memory kernel and the random force were decomposed into the radial and perpendicular contributions. The resulting EoM had the form of a DPD equation, with a transverse friction^44^ term in addition to the standard DPD friction term. This CG model could well capture the mean-square displacement (MSD), the diffusion coefficient, and the VACF of the FG system, except in the case of high *R*_*g*_ and high density where many-body correlations are important. In these cases, the Markovian assumption was also found to be inaccurate due to the lack of a clear time-scale separation.

In their following work, Li, Karniadakis, and co-workers^33^ studied melts of star polymers with CG centers at the corresponding CoM. Based on unconstrained MD simulation, they derived various DPD models with increasing degree of complexity: from the standard parametrized DPD model to DPD with radial and transverse forces and frictions and finally DPD with interactions in all three spatial directions that include explicit rotational motion of the CG particles. According to their findings, the absence of transverse interaction at the CG level leads to an underestimation of friction, whereas including it leads to an overestimation in the absence of rotational motion. When the rotation of the CG particles was accounted for in the presence of spatially resolved interactions, the DPD model could reproduce both the short- and long-time dynamics of the system. As one might expect, all DPD models except for the standard one were able to reproduce the static structure of the FG system in terms of the RDF. Yet again, the results were most satisfactory in cases where the many-body correlations could be neglected and the Markovian assumption is valid, i.e., star polymers with short arms at low density.

With an aim to extend the conditional reversible work (CRW) model^45,46^ to retain dynamical properties, Deichmann et al.^35^ used a Markovian DPD approach to coarse-grain a set of model molecular liquids, where the dissipative interactions were obtained using constrained simulations.^31,32,34^ Neopentane, tetrachloromethane, and cyclohexane were coarse-grained into a single interaction site each, with centers at their respective CoMs, and a two-site mapping was chosen for *n*-hexane. Based on the integral of the FACF, they showed that the Markovian assumption was most inaccurate in the case of *n*-hexane, where the orientation of the CG *n*-hexane was a slow DoF explicitly present at the CG level. For this system, the radial and transverse frictions were found to be comparable, similar to Trément et al.,^34^ whereas in the other three cases the latter was insignificant. The resulting dynamics in the CRW-DPD simulations showed varying accuracy in comparison to the FG results. The diffusion coefficients of all molecules, except neopentane, were found to be smaller than their FG counterparts when both the radial and transverse frictions were used, mainly due to the overestimation of the friction as previously observed by Lei et al.^32^ In the case of neopentane, however, the agreement with the FG result was very good. As we will discuss later, one possible reason for the varying performance could be the imposed constraints,^47^ which affect the dynamics of these molecules to different extents. Nonetheless, the work of Deichmann et al. highlighted the issues of long-time tails in the FACF and the lack of time-scale separation in molecular models that involve a small to medium degree of coarse-graining and multiple CG sites. These factors are relevant in chemical specific coarse-graining of polymers, where the time scales of the FG and CG systems may not be well separated.

Lemarchand et al.^36^ employed the framework of Hijón et al.^31^ to coarse-grain *cis*- and *trans*-1,4-polybutadiene and investigated the validity of the underlying Markovian and pairwise interaction assumptions. They systematically studied the effect of the degree of coarse-graining (λ) on the ability of the CG simulation to reproduce the correct dynamical and structural properties of the FG system. They observed that the dynamical properties improved with λ, owing to the better separation of the CG and FG time scales and, thereby, the accuracy of the Markovian assumption. However, the structural properties were found to deviate from those of the FG system with increasing λ due to the presence of many-body effects. Their study also highlighted the effect of constraints on the CG dynamics, where the slow rotation of the CG beads leads to a slower decay of the FACF, an artifact that is not present in unconstrained FG trajectories and had also been observed in previous studies.^35^

**Figure 1:**
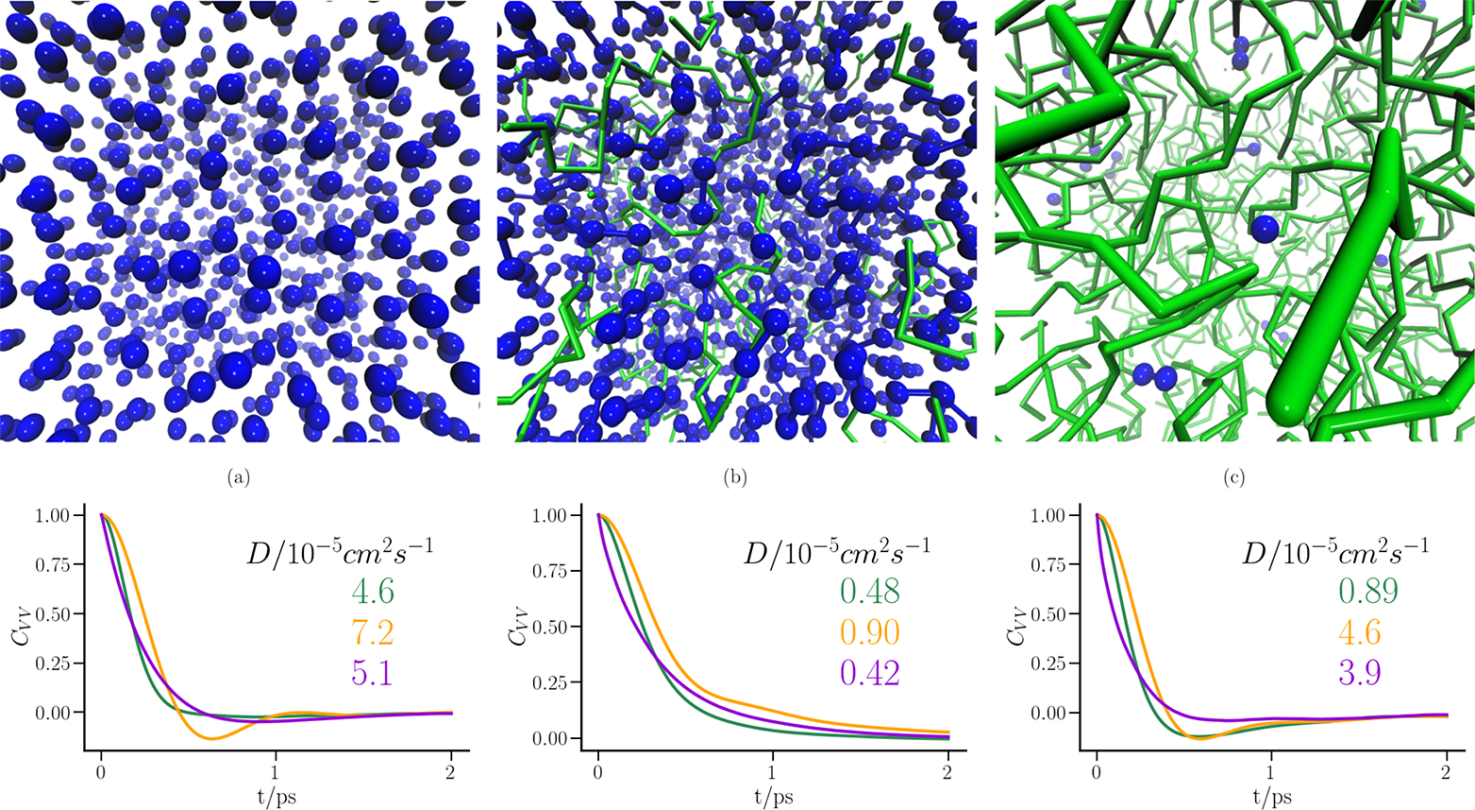
VACFs calculated using FG-MD, CG-MD, and MZ-DPD for various poly(2,2-dimethylpropane) systems: VACFs of the CoM of (a) monomers in a single-component system, (b) 24mers in 25% 24mer–75% dimer solution, and (c) monomers in a network of long poly(2,2-dimethylpropane) chains. The insets compare the corresponding diffusion constants. The top panel shows a representative configuration from each system where monomers are shown as blue beads and 24mers are shown as green chains. In spite of the apparent differences in the VACFs at shorter times, the long-time diffusion constants are better reproduced with MZ-DPD than CG-DPD in the first two cases. The CG-MD and MZ-DPD models fail to reproduce the FG-MD monomer diffusion coefficient in the polymer network. Adapted with permission from ref (37). Copyright 2018 AIP Publishing.

In their following work, Deichmann and van der Vegt^37^ performed MZ-DPD simulations of liquids, polymer solutions, and melts, comprising single- and multiple-site CG models of monomers, dimers, and 24mers based on 2,2-dimethylpropane repeat units. They used the effective-force coarse-graining (EF-CG) method^48^ to extract the conservative interactions, which also included bonded potentials in the case of the dimer and 24mer. The Q-approximation^28,32^ was employed to calculate the frictional forces from the FACF.^31^ They observed long-time tails in the FACFs, which were noticeable in the dimer case and most significant for the 24mer case. These were attributed to the slow rotation of the CG beads which led to a nonzero average fluctuating force on short time scales. The study, thus, highlighted one of the major challenges in multiple-bead representations of small molecules and polymers: Constraining the slow DoF by means of introducing bond connectivity in CG models also slows down the relaxation of the intramolecular DoF of the chemical repeat unit removed upon coarse-graining. The long tails were *a posteriori* fitted to linear functions and subsequently subtracted from the original FACFs, resulting in converging integrals. However, as shown in Figure 1, the authors reported noticeable differences between the FG-MD and MZ-DPD VACF for all of the systems under study. At short times, the particle motion is ballistic in FG-MD and dissipative in MZ-DPD, leading to faster decay of the VACF in the latter. On the other hand, elastic collisions of particles lead to a faster decay of the VACF in FG-MD at longer times. The resulting diffusion coefficients were however in good agreement with those calculated from the atomistic MD simulation of the pure liquids of monomers (see the inset of Figure 1a) and dimers. The MZ-DPD model was also found to describe polymer diffusion in polymer solutions (mixtures of dimers and 24mers), especially at low polymer density, in good agreement with FG-MD, as shown in the inset of Figure 1b. Finally, the authors investigated the dynamics of penetrants (monomers and dimers) in networks of long poly(2,2-dimethylpropane) chains in MZ-DPD. As shown in Figure 1c, the resulting long-time dynamics in this case was found to be inconsistent with the FG-MD results. The authors concluded that, in the case of molecular liquids or polymer solutions, where particle collisions govern their dynamics, the Markovian MZ-DPD approach satisfactorily reproduces the dynamics of the FG system on long time scales, in spite of the deviations at short time scales (as apparent in the VACF). However, when many-body contributions are important (the case of polymer solutions at high polymer concentration) or the dynamics is governed by activated barrier crossing^47,49,50^ (the case of penetrant diffusion in a polymer matrix), the explicit inclusion of memory effects becomes necessary.

These studies, while exploring the viability of the Markovian assumption in molecular coarse-graining, also highlight its limitations. In spite of the relative simplicity, its application has so far been mostly limited to model systems with high degrees of coarse-graining, such as LJ clusters and star polymers at low density, where the Markovian approximation remains relatively accurate. However, this approximation breaks down in cases where chemically specific CG models are used with small to medium levels of coarse-graining. The results of Trément et al.,^34^ Deichmann et al.,^35^ and Lemarchand et al.^36^ have emphasized this point. Despite incomplete time scale separations, the dynamic properties of chemically specific models could however be improved:^37^ Contrary to standard DPD with soft conservative interactions, it was demonstrated that MZ-DPD can be used to serve as a bottom-up-informed thermostat that fixes the long-time diffusive dynamics in the coarse-grained simulations of molecular liquids in which hard-core repulsions are retained. This work additionally emphasized the need to incorporate memory effects in the CG model when the dynamics is governed by activated barrier crossing as opposed to particle collisions as in molecular liquids.

## 4 Reconstruction of Memory Kernels

While the original MZ theory was developed already in the early 1960s,^19,20^ recently, it has regained a lot of attention in the context of dynamic molecular coarse-graining, where the memory kernels are extracted from FG trajectories. As discussed in the previous section, the Q-approximation has been extensively used to parametrize CG DPD models of chemical systems with varying success^31,34,37^ and the limitations have also been discussed. Recently, attempts are also being made to find solutions for the plateau problem.^51^ Nonetheless, the most straightforward way to calculate friction coefficients is to formulate an appropriate GLE for the system under consideration, from which methods for the extraction of the memory kernel can be developed. This not only allows a more accurate determination of friction coefficients but also enables the study of time- or frequency-dependent phenomena based on the memory kernel. In the case of low-dimensional GLEs, e.g., GLEs for single diffusing particles, it is possible to exactly reconstruct memory kernels (within numerical and statistical errors) from FG simulation trajectories. Several methods have been developed, some of which are reviewed in this section.

We begin with some general remarks. A typical problem in memory reconstruction is to determine memory kernels from a given auto-correlation function *C*_*AA*_(*t*) = ⟨*A*(0)*A*(*t*)⟩ of a target CG observable *A* that is taken to evolve according to a GLE, eq 5. Multiplying eq 5 with *A*(0) and taking the thermal average, one derives an equation for *C*_*AA*_(*t*)
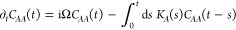
18In the case Ω = 0, eq 18 has the form of a Volterra equation of the first kind. It can be inverted numerically, e.g., by Laplace transform. However, from the point of view of numerical stability, it is often more convenient to first take the time derivative, thus converting eq 18 into a Volterra equation of the second kind^52^
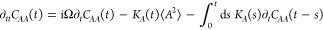
19for which more stable algorithms exist. We note that the time derivatives ∂_*t*_*C*_*AA*_(*t*) = *C*_*ȦA*_(*t*) and ∂_*tt*_*C*_*AA*_(*t*) = −*C*_*ȦȦ*_(*t*) can often be determined directly from simulations, so that it is not necessary to numerically calculate the derivatives of *C*_*AA*_(*t*).

Alternatively, one can also integrate eq 18,^53−55^ which yields an equation for the running integral over the memory kernel: *G*_*A*_(*t*) =  d*s* *K*_*A*_(*s*),
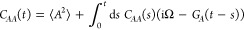
20Replacing the origin of time *t* = 0 by *t* = *t*_0_ throughout and taking the derivative with respect to *t*_0_ for *t*_0_ → 0, one can derive an implicit equation^54^ for the quantity *J*_*A*_(*t*) = i Ω – *G*_*A*_(*t*):
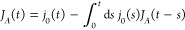
21with . It can either be solved directly by matrix inversion after discretization in time^55^ or iteratively^54^ by successive application of eq 21
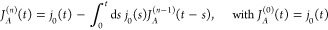
22This method can also be used to determine memory kernels *K*(*t*, *t*_0_) in nonstationary nonequilibrium situations.^54^ In that case, eq 21 for *J*_*A*_(*t*, *t*_0_) = iΩ(*t*) –  d*s* *K*_*A*_(*s*, *t*_0_) reads
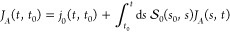
23with 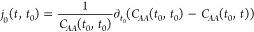 and 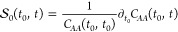


The methods described above have been developed for linear GLEs and cannot easily be extended to GLEs that contain anharmonic conservative force terms (as may occur in eq 12). In that case, numerical reconstruction methods can be applied that rely on an iterative refinement of *K*_*A*_(*t*) based on successive GLE simulations,^56,57^ similar to the iterative Boltzmann inversion (IBI) method in structural coarse-graining.^58^

In the next sections, we will now present specific examples of memory reconstruction methods for low-dimensional GLEs. In multidimensional systems, e.g., multiparticle systems, further approximations are necessary, which are mainly discussed in section 5.

### 4.1 Freely Diffusing Particles

In the simplest case of freely diffusing particles, the EoM of a system can be formulated in terms of a GLE without any conservative interactions. For simplicity, we will consider one-dimensional systems. The GLE then takes the form
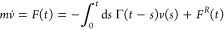
24It describes the CoM dynamics of a tagged particle with velocity *v* in an isotropic solvent. As discussed earlier, in the limit of large particle mass, eq 24 can be reduced to a Markovian LE, which describes the motion of a heavy Brownian particle. In the Markovian case, the dynamics is governed by the scalar friction coefficient γ, which determines the diffusion coefficient via the Stokes–Einstein relation and leads to a VACF that shows an exponential decay and determines the MSD. In a similar way, the memory kernel Γ(*t*) determines the dynamics of a single tagged particle with memory. According to eq 20, the VACF obeys the relation
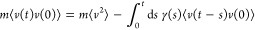
25where γ(*s*) =  d*s*′ Γ(*s*′). Using ⟨*Δx*^2^(*t*)⟩ =  d*t*′  d*t*″⟨*v*(*t*′)*v*(*t*″)⟩ and the equipartition relation *m*⟨*v*^2^⟩ = *k*_B_*T*, one can derive an equation for the mean-square displacement (MSD)^53^
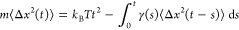
26On long time scales, once the memory function has fully decayed, the dynamics becomes uncorrelated, thus fulfilling the Stokes–Einstein relation. The friction coefficient governing the diffusion on long time scales is then given by γ =  Γ(*t*) d*t*. The MSD for a memoryless LE exhibits a ballistic regime at time scales *t* ≈ 0 and smoothly transitions into a linear regime for larger time scales. Anomalous diffusion with different scaling exponents can thus be attributed to the memory kernel, as given by eq 26. It is known that subdiffusive dynamics, in which the MSD scales as ⟨*Δx*^2^(*t*)⟩ ∝ *t*^α^ with α < 1, can be described in terms of a GLE with a memory kernel of the form Γ(*t*) ∝ *t*^–α^ at large times.^59^ This especially occurs in viscoelastic materials such as polymer melts, in which stresses relax very slowly.

Over the last couple of decades, different methods have been proposed to extract the memory kernel of a tagged particle from trajectories based on higher resolution (FG) models.^52,53,56,60−66^ One approach is to discretize eq 25 or 26, calculate γ(*t*) from the time evolution of the position of a tagged particle,^53^ and then take the time derivative. Another widely used approach^29,52,67^ is based on the Volterra eqs 18 and 19, which here can be written in the form
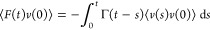
27and
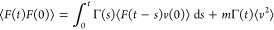
28The force–velocity correlation function (FVCF) and the FACF can be computed directly from the FG trajectories. Subsequently, Γ(*t*) can be calculated from eq 28 by discretization in the time domain^52,67,68^ or by exploiting the convolution theorem to extract Γ(*t*) in the Fourier or Laplace space.^27,63,69−71^ Additional relations can be formulated in the Fourier space such as
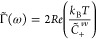
29and^72^
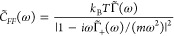
30where *C̃*_+_^*vv*^ is the one-sided Fourier transform of the VACF and *C̃*_*FF*_(ω) is the Fourier transform of the FACF.

While Γ(*t*) can be obtained from eq 29 by means of an inverse Fourier transform, eq 30 can be solved by assuming a functional form of Γ(*t*) and optimizing the fitting parameters, which reproduce *C̃*_*FF*_(ω).^72^ Kowalik et al.^53^ compared the performance of approaches derived from eqs 25–30 for calculating the memory kernel of a freely diffusing methane particle in water. The authors found that the methods described by eqs 25, 26, 29, and 30 perform equally well, while methods based on eqs 27 and 28 are prone to numerical instabilities at long times. In general, the high-frequency contributions of the memory kernel are usually better reconstructed by methods that are directly based on the force auto-correlation function, while discretization errors in the long-time dynamics can commonly be reduced using slower decaying correlation functions such as the VACF. Recently, this observation has been used to construct a high-precision hybrid method.^73^

While the memory kernel at thermal equilibrium can be described in terms of the FDT, *k*_B_*T Γ*(*t*) = ⟨*F*^*R*^(*t*)*F*^*R*^(0)⟩, the above-mentioned methods to extract the memory kernel do not require the direct calculation of the projected dynamics defined in the MZ formalism. They rather exploit general properties of the GLE which are independent of its MZ theory background. Carof et al. derived a method to explicitly calculate the projected force correlation function from the FG trajectories based on a rigorous application of the MZ theory.^60^ The original numerical schemes applied first order approximations for numerical discretizations, while second order schemes were shown to be significantly more accurate.^56,61^ While the extracted memory kernels should be the same as those obtained with the other methods discussed above (within the numerical error), the projected dynamics scheme by Carof et al. offers more general insight, as it also allows one to calculate the projected dynamics for other dynamical variables that depend on the chosen CG variables. This allows, for example, one to separate interactions into different contributions and independently calculate their contributions to the memory kernel and, thus, to the total friction. This was applied in the same study to calculate the contributions of short-range repulsive and long-range attractive interactions and their cross-correlations to the memory kernel. Based on their results, the authors concluded that friction in LJ fluids is dominated by the short-range interactions, which is expected, as the repulsive interactions are much steeper and thus contribute to dissipation through a stronger transfer of momentum.

Recently, two works have explored the possibility of using fine-grained trajectories to extract extended Markov models^74,75^ from which the memory kernel can be calculated. The idea of extended Markov models is to artificially include a coupling of the CG variables to additional degrees of freedom with Markovian interactions, which mimic the non-Markovian dynamics of the system. This approach thus directly combines reconstruction of memory with the construction of models that can be integrated very efficiently, as will be discussed in detail in section 6.2.

### 4.2 Particles Diffusing in Harmonic Potentials

Studies of particles diffusing in harmonic potentials are of special interest, because such potentials can model typical setups of single-molecule force spectroscopy and/or microrheological experiments. In such experiments, optical or magnetic tweezers are used to trap large molecules such as DNA, proteins, or colloids. The tweezers can be calibrated such that, effectively, a harmonic external potential is applied to the trapped tracer particle. Monitoring the trajectory allows one to calculate the rheological properties of the fluid in which they are suspended. However, the temporal resolution in experiments is typically limited to a time scale of ∼0.1 ms, which is too large to resolve atomistic fluctuations; therefore, an interpretation in terms of GLEs is appropriate.

In the analysis of experimental data, the motion is typically taken to be overdamped. If the mass of the tracer particles is large, memory effects can be neglected. This approximation is well justified for tracer particles of size around ∼0.25–0.5 μm.^76^ The standard procedure in the analysis of force spectroscopy measurements is thus to fit the power spectrum of positional noise by a Lorentzian function, from which the viscosity of the fluid can be deduced. Taking memory effects into account in the analysis of the experimental data can give further information on the properties of the fluid. For example, the measurement of the frequency-dependent viscosity gives insight into the viscoelastic properties such as the storage and the loss moduli.^77^ In order to understand such experiments, one must understand the effect of confinement on the measured rheological properties.

Daldrop et al.^72^ and Kowalik et al.^53^ have studied memory effects of solutes whose CG EoM is given by the GLE
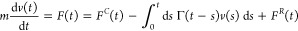
31where *F*^*C*^(*t*) is the force due to an external harmonic potential, *F*^*C*^(*t*) = *kx*(*t*). The case *k* = 0 describes a freely diffusing particle and the case *k* = ∞ can be implemented by constrained dynamics. In ref (72), the authors carried out atomistic MD simulations of a single methane molecule in water, wherein a harmonic confinement potential was applied to the CoM of the molecule. To extract the memory kernel, they derived a generalized variant of eq 30
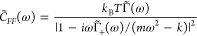
32from which the friction coefficient for *k* ≠ 0 can be evaluated as
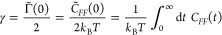
33Here Γ̃(0) and *C̃*_*FF*_(0) are the Fourier transforms of the memory kernel and the FACF at frequency ω = 0, which can be evaluated as the time integrals over Γ(*t*) and *C*_*FF*_(*t*). Equation 33 shows that the friction coefficient can be extracted directly from the integral of the FACF for weak confinement forces. As mentioned in section 3, this is not possible for unconfined dynamics due to the plateau problem. By varying the strength *k* of the confining potential, its influence on the friction coefficient can be evaluated. It is important to stress that eq 33 only holds for the frequency ω = 0 and thus only relates the integrals of the memory kernel and the FACF, but not the functional form itself.

Daldrop et al.^72^ analyzed the influence of the confinement on the form of the FACF and the memory kernel independently. For weak confinement, the integral over the FACF exhibits a distinct maximum value followed by a decay to zero similar to the unconfined case. On larger time scales, the weak confining forces induce a long-lived positive tail in the FACF which generates a finite plateau in the running integral over the FACF on large time scales. Harmonic potentials were shown to slow down the relaxation of the FACF on intermediate time scales. This leads to an increase in the plateau value of the integral in confined simulations and thus to an increase in the apparent friction coefficient. In the limiting case of a constrained particle, the friction coefficient was found to be overestimated by a factor of ∼1.5. The authors note that this enhancement of the friction due to confinement does not result from any structural changes in the solvation shell, as the confinement forces do not affect the equilibrium structural properties. However, the confinement of the methane molecule influences the relaxation of the water molecules in the hydration shell, effectively increasing the local viscosity in the first hydration shell. They observed a similar effect when artificially increasing the mass of the methane molecule.^78^ Higher solute masses also resulted in a slowdown of hydration shell dynamics and a local increase of the viscosity.

In the above approach, the memory kernel Γ(*t*) was extracted by parametrization, which allowed a separation of contributions to the memory kernel on different time scales. The authors could attribute them to distinct molecular processes^72^ and concluded that the imposed confinement mainly affects the hydrogen bond breaking processes. The time-scale analysis furthermore suggested that the impact of confinement on the local viscosity is only significant if the inertial time scale of the tagged particle is comparable to or smaller than the time scale of the memory kernel. In the Markovian limit of heavy particles, confinement is not expected to influence the measured friction.

In a follow-up study,^53^ the authors studied the influence of harmonic potentials on the memory kernel for a broader set of solutes and solvents with varying viscosities. The solutes under study were methane, water, sodium cations, sodium anions, and glycerol, while the viscosity of the solvent was varied by changing the composition of a water–glycerol mixture. When comparing different solutes for a fixed solvent, the confinement effects on the friction were found to be negatively correlated with the amplitude of the friction coefficient of the free solute. On the other hand, when varying the solvent for a fixed solute (i.e., a confined glycerol molecule), the correlation was positive. This can be understood in terms of time-scale separation due to size effects: The larger the solute and the less viscous the solvent, the clearer is the time-scale separation and, hence, the smaller the memory-induced confinement effects on the friction.

As mentioned above, the computational studies of Daldrop and Kowalik et al.^53,72^ can give insight into the dynamical processes in typical single-molecule force spectroscopy experiments. The numerical findings^53^ suggest that significant confinement effects are unlikely in typical optical trap experiments, as the applied harmonic potentials are too weak and thus introduce modes which have larger time scales than the memory kernel. However, the spring constants applied in atomic force microscopy experiments can be orders of magnitude higher and thus can couple with the dynamical modes of the solvent, thereby introducing confinement-dependent frictional effects.

### 4.3 Iterative Reconstruction

The memory reconstruction methods described above are restricted to freely diffusing particles and particles in harmonic potentials. Jung et al. introduced two techniques for the iterative reconstruction of memory kernels (IMR) from FG simulations,^56^ which can be applied more generally.

The methods take their inspiration from the iterative Boltzmann inversion (IBI) method, which was introduced for structural coarse-graining.^58^ The memory reconstruction methods use either the force correlation function (IMRF) or the velocity correlation function (IMRV) as the target function in the iterative schemes. The IMRF method is based on the fact that in the infinite mass limit the force correlation function is exactly proportional to the memory kernel. This can be used to motivate an iterative optimization scheme for the memory kernel which is linear in the deviations of the force correlation functions determined from the FG input and CG simulations using the current guess for the memory kernel. The iterative procedure is initialized using the Q-approximation; i.e., the memory kernel is initialized as the FACF. Starting from the IMRF method, the IMRV method exploits the fact that the second derivative of the VACF is proportional to the FACF; hence, the FACF is replaced by the finite-difference representation of the second derivative of the VACF in the IMRV scheme. To enhance convergence of the optimization procedure, a time-dependent and adaptive choice for the step size of any given iteration was introduced.

The method was evaluated using the example of a freely diffusing colloid in a LJ particle bath. Both IMRV and IMRF were applied for the reconstruction of the memory kernel starting with the FACF as the initial guess. Both schemes reasonably converged after 100 iterations. The IMRV was found to be more stable, i.e., exhibiting less noise in the resulting memory kernel, and resulted in a better representation of the VACF in the final model. The memory kernel obtained by the IMRV was also compared to the memory kernel as calculated from inverting the Volterra equation (eq 27) or determining the projected force correlation function following Carof et al.,^60^ and the results were found to be virtually equivalent. In terms of reproducing the VACF of the underlying system, the IMRV scheme, by construction, proved to be less prone to errors due to discretization. Moreover, the IMRV method optimizes, also by construction, the representation of the memory kernel in the target GLE integration scheme, and thus automatically accounts for time-discretization effects at the GLE level. In the example above, the time step in the GLE simulations could be chosen to be 200 times larger than that in the FG simulations, making the integration of the GLE efficient, despite the need of explicitly calculating the convolution integral (see also section 6). In a follow-up paper, Jung et al. applied their method to the reconstruction of pair memory kernels.^57^ This work will be discussed in more detail in section 5.

The recent work by Wang et al.^74^ is based on a similar iterative approach and optimizes the CG model via a Bayesian optimization scheme.

### 4.4 Generalized Variables

The Mori–Zwanzig formalism and the memory reconstruction methods quoted above are clearly not restricted to particle-based descriptions but can similarly be applied to generalized coordinates. Some popular examples are molecular hydrodynamic or fluctuating hydrodynamic descriptions,^79−82^ in which the distinguished variables are density, energy density, and longitudinal current modes and the corresponding correlation functions are, e.g., intermediate scattering functions (ISFs). In this subsection, we will briefly discuss such techniques.

Deriving molecular hydrodynamic equations is one of the oldest applications of the memory function formalism.^79,80^ Originally, it was believed that certain correlation functions (i.e., the VACF) must decay exponentially in time due to the molecular chaos assumption, which states that collisions experienced by a particle in a fluid are uncorrelated. However, in a pioneering work in the 1970s, Alder and Wainwright unmistakably demonstrated the existence of long-time tails already in hard-sphere fluids.^83^ Their observation could be explained based on a molecular hydrodynamic description, in which the memory kernel is approximated using mode-coupling theory.^84^ Similar anomalous properties of various important transport coefficients have been studied extensively since then, also in the context of the glass transition.^85^ For detailed discussions, we refer to recent reviews and standard textbooks on related topics such as anomalous transport,^86^ molecular hydrodynamics,^80^ and memory in glassy systems.^85,87,88^

Amati et al.^89,90^ used the Mori–Zwanzig formalism to study memory effects in the density fluctuations of a Fermi–Pasta–Ulam model, i.e., a linear chain with anharmonic bond potential. The reconstruction technique was based on a series expansion of the numerically calculated ISF. The detailed analysis of the short-time behavior of both the classical and quantum mechanical versions of the Fermi–Pasta–Ulam model revealed zero-point energy effects that affect the mobility of the particles.

Chen et al. investigated the non-Markovian conformational motion of large proteins such as HIV-1 protease, which consists of nearly 200 residues,^91^ showing that the conformational motion of proteins, which is usually modeled via Markov models, can exhibit memory effects, depending on the degree of coarse-graining. This study was based on an analysis of the potential energy of the protein only and did not yet include solvent effects. Later, Ma et al.^92^ and Lee et al.^93^ used molecular simulations to reconstruct the non-Markovian conformational motion of chignolin^92^ and alanine dipeptide.^93^

Memory kernels have also been reconstructed for nonequilibrium nonstationary GLEs.^24^ Meyer et al. used their memory reconstruction methods (eq 22) to study the fundamental problem of nucleation.^54,94^ In this case, the time dependence of the nucleation-cluster size was chosen to be the relevant generalized variable. The authors found intriguing non-Markovian effects in the dynamics of the cluster size, which explicitly depend on the age of the sample.

## 5 GLE-Based Coarse-Graining and Multiscale Modeling

In the previous section, we have discussed how FG systems can be mapped onto (mostly low-dimensional) GLEs in order to study the nonlocal effects in the friction (memory kernel) and properties of colored noise. In dynamic coarse-graining, the goal is often to construct dynamically consistent high-dimensional CG models with many interacting CG variables. Such efforts will be discussed in this section.

Smith et al.^95,96^ and Tuckerman et al.^97^ were among the first to derive an effective GLE type EoM from MD simulations and employ it in CG simulations. While the foundations of this approach were thus already laid quite some time ago, in recent years, increasing efforts have been dedicated to deriving methods for non-Markovian CG models using bottom-up approaches. So far, successful models in this direction include models on freely diffusing Brownian particles with single-particle friction kernels,^29,67^ dilute and dense particle systems with pairwise friction interactions,^29,57,98,99^ and also models based on generalized CG variables that do not have a (CG) particle interpretation such as density fields.^100,101^

### 5.1 Particle-Based Coarse-Graining

The earliest attempts to solve stochastic differential equations with interactions that are nonlocal in time date back to the beginning of the 1980s with the works of Ermak and Buckholz^102^ and Ciccotti and Ryckaert.^103^ Details of the numerical implementations will be discussed in section 6. Smith et al.^95,96^ were the first to apply these ideas to real systems and to thus propose a systematic dynamic coarse-graining procedure. They applied their methods to the vibrational relaxation of iodine suspended in LJ xenon at *T* = 300 K. The integration of the generalized Langevin equation is based on an auto-regression model, which has been shown to be equivalent to the method of Ciccotti and Ryckaert^103^ and related to the auxiliary variable approaches discussed in section 6.2. They compared the results of their GLE model to MD simulations, showing that such a simple model is indeed able to describe the FG dynamics in full detail, thus laying the foundation for future works on dynamic coarse-graining. One year later, Tuckerman and Berne^97^ used methods derived earlier by Berne et al.^65,66^ to extract the memory kernel of a constrained diatomic LJ harmonic oscillator immersed in a LJ particle bath. Later, they generalized this to anharmonic coupling,^104^ thus providing the first dynamically consistent coarse-grained model in a complex energy landscape.

Only recently, this idea was brought back to life and generalized to multiparticle systems. The simplest approach is to neglect particle correlations in the friction terms and assume that the motion of CG particles can be described by a single effective “self-friction kernel” according to the EoM^29,67,105^
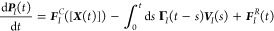
34where **Γ**(*t*) is a single-particle memory kernel and particles can only interact via the conservative forces ***F***_*I*_^*C*^([***X***(*t*)]).

Recently, Wang et al.^74^ showed that, for star polymer systems, eq 34 suffices to reproduce dynamical properties of the underlying FG system over density ranges from dilute solutions to a melt. In this study, all memory effects were described by an average scalar self-friction memory kernel, which can be modeled by the auxiliary variable approach (see section 6.2). The authors used a Gaussian process based Bayesian optimization scheme^106^ to optimize the memory kernel to match the VACF of a single particle. The fundamental idea is comparable to the IMRV scheme; however, it is better suited for the auxiliary variable approach, because the parameters of the integrator are optimized directly instead of being fitted *a posteriori* to a memory kernel. A similar Bayesian approach was used to parametrize CG DPD models in ref (107).

While these models can well reproduce the tagged-particle motion, it is expected that pair diffusion will not be appropriately described. Already in 1990, Straub et al. showed that the relative motion between two bounded LJ particles can be described by a GLE with a memory kernel that strongly depends on the particle distance.^108^ An alternative approach is thus to assume that the friction forces can be decomposed into pair friction terms that solely depend on the relative velocity ***V***_*IJ*_ of the interacting particles *I* and *J*,^29,108^ resulting in the approximation (cf. eq 14)
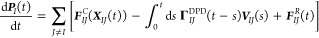
35As discussed in section 2, this corresponds to a non-Markovian extension of DPD-like models. For such models, an additional fundamental problem arises: Pair memory kernels typically depend on the distance between particles, which changes with time. Therefore, the problem of determining pair frictions is only well-defined in cases where the distance between the particles is confined by a potential, e.g., a bond potential, or if the CG sites belong to the same molecule.^108^ In all other cases, one must make the additional approximation that the particle distance is roughly constant on the time scale of memory decay; i.e., one must assume that the time scales of the memory kernel and the characteristic diffusion time of particles are well separated. If this is indeed the case, pair memory kernels can be extracted from FG simulations in the same way as single-particle memory kernels^29,108,109^ (section 4).

Li et al. considered a GLE of the form of eq 35 and introduced a pairwise decomposition of conservative interaction and the memory kernel.^29,98,109^ The EF-CG approach^48^ and the IBI^58^ method were used to derive the conservative interactions, while a pairwise variant of the Volterra equation (eq 27) was used for the derivation of the pairwise memory kernels. Furthermore, for numerical simplicity, the time and distance dependence of the memory kernels were assumed to be separable. In all cases, the star polymer systems were considered with varying polymer sizes and densities.

**Figure 2:**
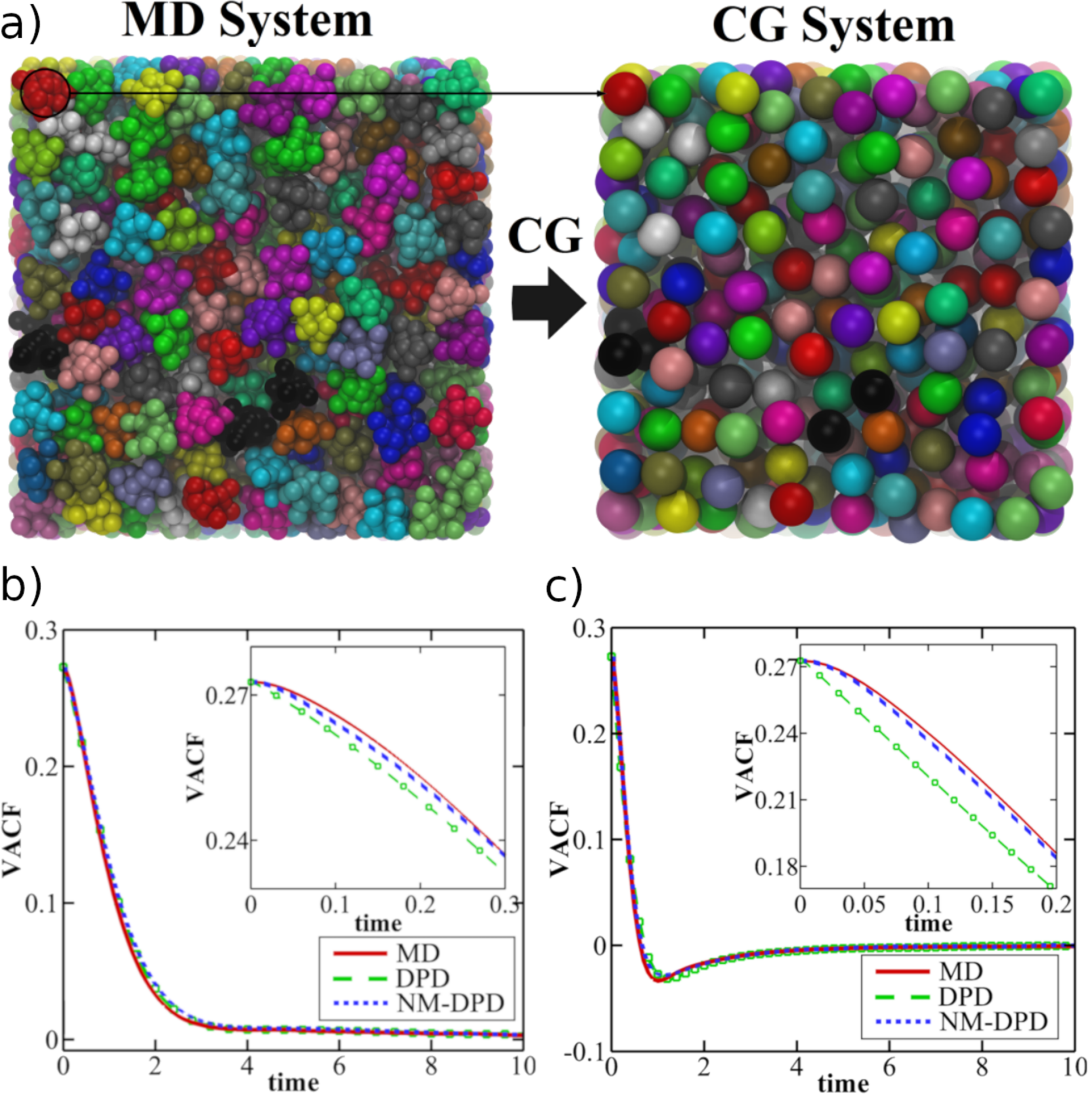
Non-Markovian coarse-graining procedure for a star polymer melt.^98^ (a) Illustration of the coarse-graining procedure in which each polymer is replaced by a single CG particle, which interacts with the other particles via the EoM (35). (b, c) VACF for the non-Markovian DPD (NM-DPD) model in comparison to the MD results and a Markovian DPD model for low (b) and high (c) density. Reprinted with permission from ref (98). Copyright 2015 AIP Publishing.

In ref (98), Li et al. considered star polymers consisting of 11 beads interacting through Weeks–Chandler–Andersen interactions at reduced densities of 0.4 and 0.7. They found that, at both densities, the non-Markovian DPD approach performed well in reproducing the VACF of the underlying FG system (see Figure 2). A comparison with Markovian DPD simulations further showed that the improvement due to the incorporation of memory effects was stronger for the dense systems, which lacked time-scale separation. However, the Markovian DPD simulations also performed relatively well at both densities, which highlighted the possibility of using Markovian approximations in a wide range of implicit solvent polymer systems, depending on the desired accuracy. Only for high frequencies (i.e., small times), one can observe clear deviations between the non-Markovian and Markovian DPD models, as highlighted in the insets in Figure 2.

Yoshimoto et al.^109^ combined a non-Markovian DPD model with the IBI^58^ and EF-CG^48^ methods and applied it to a dense system of LJ colloids. They found that the dynamic properties did not depend on the specific coarse-graining strategy for the conservative interactions. Furthermore, they compared two different approaches for extracting the memory kernel: first, approximating the memory kernel by the force auto-correlation function (Q-approximation), and second, by inverting the Volterra equation. Since the chosen system was dense, a time-scale separation cannot be assumed and the memory kernel extracted from the Volterra equation led to a better representation of the dynamics. Being exact for *t* = 0, the Q-approximation shows good agreement for the short-time behavior; however, for long times, the force auto-correlation function significantly deviates from the real memory kernel and also suffers from the plateau problem,^38,40^ as discussed earlier.

Another interesting, more qualitative approach to include memory on the pairwise level to coarse-grained simulations has been suggested in ref (110) and applied several times since then in the context of star polymer melts^111^ and polymer solutions.^112^ The idea is to include additional, physically motivated degrees of freedom to the system which mimic the slow structural relaxation of the orthogonal variables. This approach is thus connected to the data-driven auxiliary variable approach, in which these additional degrees of freedom, however, usually do not have any physical interpretation.

The “pure self-friction kernel” models (eq 34) and the non-Markovian DPD models (eq 35) discussed so far can be implemented efficiently, but they impose rather severe restrictions on the form of the multiparticle memory kernel, compared to eq 12. Moreover, they are not even compatible with each other. In particular, the self-friction contribution of the memory kernel in the non-Markovian DPD model
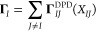
36depends solely on the surrounding particles and may either become very large (in dense systems) or very small (in dilute systems). This causes problems, e.g., when looking at colloidal suspensions where the dominant friction stems from the interaction with the (implicit) solvent, but collective memory effects^113^ (frequency-dependent hydrodynamic interactions) may, nevertheless, not be neglected. Theoretical and numerical studies of a system containing two colloids only reveal an intriguing dependence of both the pair- and self-memory on the interparticle distance.^113^ Methods that are purely based on self-memory or on DPD-type pair-friction are thus expected to fail. To solve this problem, Jung et al.^57^ proposed a generalization of the non-Markovian DPD models. In this study, the memory matrix as defined in eq 12 consists of a self-memory matrix coupling to the velocity of the particle and a set of pair matrices coupling to the velocities of the other particles in the system.
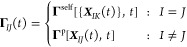
37The self-memory matrix is assumed to depend on the configuration, as the friction with respect to the background medium can be altered by nearby particles.^113^ It thus has a configuration-independent “bare” component and a contribution that depends on the relative positions of other particles in the vicinity
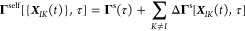
38

**Figure 3:**
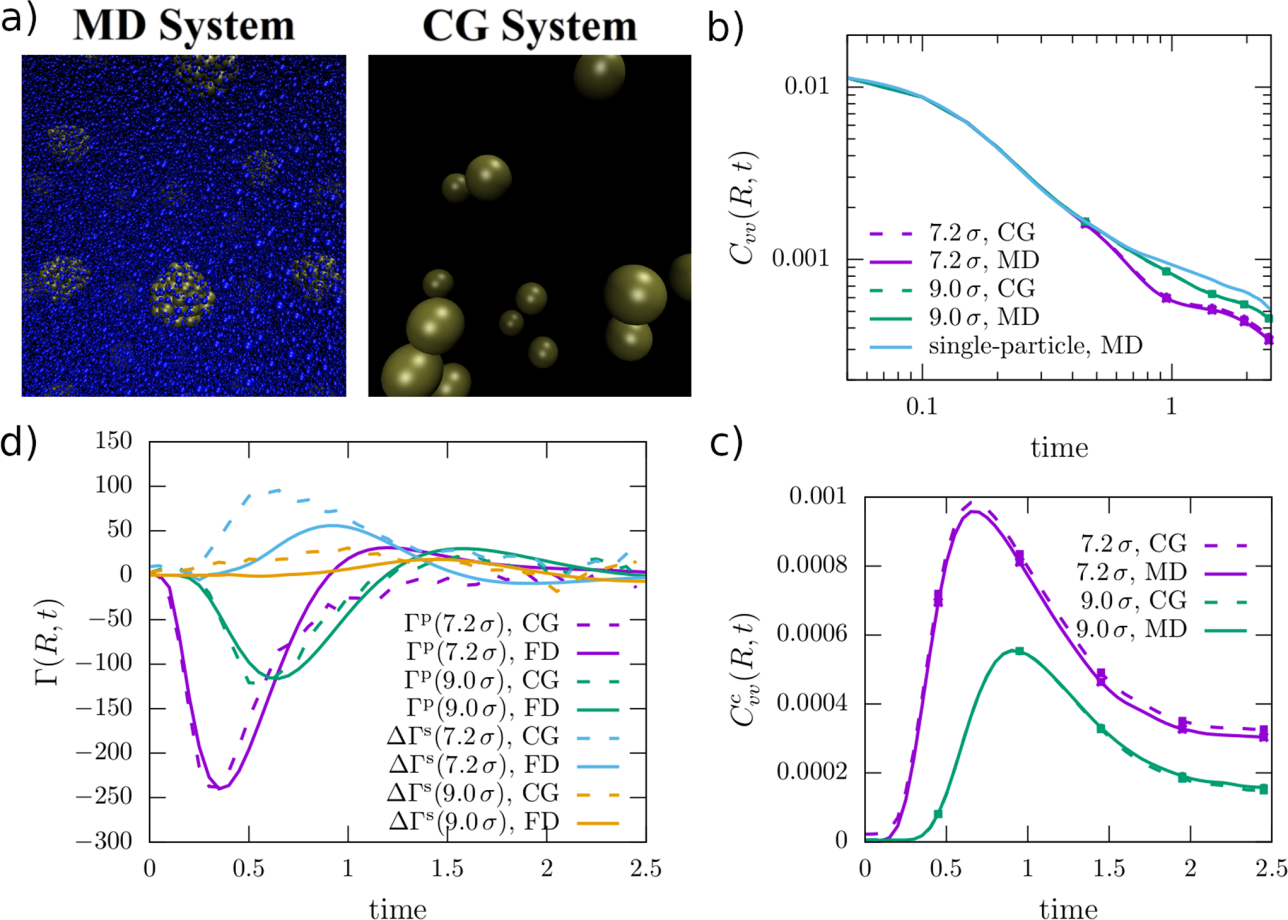
Non-Markovian coarse-graining procedure for a colloidal suspension. (a) Illustration of the coarse-graining procedure, in which every colloid is represented by a single CG particle and the interaction with the solvent is incorported purely implicitly. (b) The velocity auto-correlation function, *C*_*vv*_(*R*, *t*), for colloids which have a nearest neighbor at a distance *R*. (c) The velocity cross-correlation function, *C*_*vv*_^*c*^(*R*, *t*), for pairs of colloids at distance *R*. The results in parts b and c are compared between MD results and the non-Markovian coarse-grained model (CG). (d) Comparison of the reconstructed memory kernels Γ(*R*, *t*) of the CG model (see eqs 37 and 38) with fluid dynamics (FD) theory.^113^ This also shows the importance of the introduction of distance-dependent memory kernels. Figure adapted with permission from ref (57). Copyright 2018 Royal Society of Chemistry.

The set of eqs 37 and 38 is still less general than eq 12, but it can interpolate between eqs 34 and 35 and includes them both as special cases. Using this framework, Jung et al. studied a dilute system of repulsive nanocolloids (radius *R*_*c*_ = 3σ) in a LJ liquid (diameter *d* = 1σ), as illustrated in Figure 3. The memory kernel was reconstructed using the iterative reconstruction.^56^ As an initial guess for the memory kernel, a generalization of the Volterra equation (eq 27) including distance-dependent velocity auto- and cross-correlations for a system containing only two particles was used, similar to ref (113). Effective many-body effects in multiparticle systems were then implicitly introduced by optimizing the memory matrix via the IMRV method. In order to validate and test the approach, the authors compared the distance-dependent velocity auto-correlation and cross-correlation functions from the original FG system to those in their model, with excellent results, as shown in Figure 3b and c. The authors also compared the reconstructed memory kernel to fluid dynamics theory, obtained by analytically solving the linearized Navier–Stokes equation for two embedded spheres.^113^ The simulation and theoretical results are in quantitative agreement (see Figure 3d), which not only validates the assumptions made for the simulation model but also highlights the importance of using distance-dependent memory kernels to capture the relevant physics of the fluid. Moreover, for the first time, the authors also analyzed the *transferability* of the CG model to different colloid densities. They found that the model not only describes the dynamic properties of one particular system but indeed captures the fundamental non-Markovian interactions of colloids suspended in a Lennard-Jones fluid over a wide range of colloid densities. A significant gain in performance could be achieved for colloid number densities corresponding to dilute systems compared to FG simulations, not only due to the reduction of the number of particles but also because the time step could be chosen to be about 50 times larger than that in the reference FG simulations.

The portfolio of methods for bottom-up non-Markovian CG simulations with consistent dynamics has grown quite substantially over the past decade. The choice of the method strongly depends on the system under study and the properties of interest. The general method proposed by Jung et al.^57^ can be applied to a large set of systems and is most efficient in cases where the relevant particles only represent a very small fraction of the microscopic degrees of freedom, e.g., in implicit solvent models. In the opposite case, in which the coarse-grained system incorporates most of the microscopic degrees of freedom, as is the case, for example, for the coarse-graining of polymer melts, the non-Markovian DPD approach by Li et al.^29^ might, however, be more suitable due to its numerical efficiency. Both methods are clearly less efficient compared to the pure self-friction models that have been applied in refs (48, 98, and 105). These simplified models are able to describe tagged-particle motion in a numerically efficient and dynamically consistent manner. Many physical and chemical processes, such as hydrodynamic motion or diffusion in complex environments, however, crucially depend on the relative motion of molecules. An additional problem is the transferability of these models. Since the single-particle memory does not include any information on the (local) density of the system, one would expect that the models can only reproduce the correct dynamics in exactly the same system in which they were reconstructed and that any change of state variables will require a re-evaluation of the memory kernel. Furthermore, any information on dynamic heterogeneities in the system will be lost due to the averaging over all particles. These problems will have to be discussed in the future in order to improve the practical use of dynamically consistent coarse-grained models.

### 5.2 Coarse-Graining with Generalized Collective Variables

Much of the work on GLE-based coarse-graining so far has addressed particle-based CG models. In section 4.4, we have discussed some recent works where memory kernels were reconstructed for GLEs operating with generalized collective variables, focusing on the interpretation of memory effects in dynamics and not on the construction of CG models for actual non-Markovian simulations. In the following, we will highlight a few examples where GLE-based coarse-graining was applied to derive CG models with generalized CG variables.

One example is the set of non-Markovian models that were constructed to describe the conformational motion in proteins,^91−93^ which were already mentioned in section 4.4. Chen et al. studied a high-dimensional model, where the coarse-grained variables correspond to low-frequency eigenmodes of HIV-I protease (the authors also provided results for more standard, particle-based coarse-graining). In terms of complexity, the model operates on a similar level as ref (57), introducing dissipative forces for both the self- and pair-interactions in the system.

Other examples are the non-Markovian dynamic density functionals, which are attracting growing attention. Very recently, Russo et al.^114^ developed a multiscale framework for describing reacting multispecies fluids in equilibrium and nonequilibrium. They started from an already coarse-grained GLE-system of particles with pure self-memory, and then performed ensemble averages over local densities, momenta, and reaction sources, applying a local equilibrium assumption. The resulting theory had the form of a fluctuating non-Markovian dynamic density functional and was used to study, e.g., the diffusion of a gas in a double well potential and the influence of memory on Turing patterns.

Memory effects are particularly prominent in polymer systems where the dynamics of density fluctuations is governed by chain relaxation processes on multiple time scales.^115−117^ Wang et al.^118^ recently investigated the influence of memory on the kinetics of relaxation and structure formation in copolymer melts and polymer blends. They derived an analytic expression for the memory kernel in random-phase approximation and constructed a non-Markovian dynamic density functional theory (NM-DDFT) based on this kernel. They showed that NM-DDFT calculations can quantitatively reproduce the collective disordering dynamics of particle-based reference simulations. Based on this work, Rottler and Müller^119^ used the method of Meyer et al.^54^ (eq 22) and further approximations regarding the collective dynamic structure factor to derive a memory kernel for block copolymer melts and applied it to study pattern formation in thin block copolymer films.

Memory is also a central ingredient in the recently proposed hydrodynamic models for fluctuating viscoelasticity.^120−122^ The Oldroyd-B and related models for viscoelastic flow of polymeric melts are examples of multiscale models with memory, where the memory is approximated by a physically motivated auxiliary variable, which is usally denoted as an extension tensor that basically “memorizes” the local extension of polymers. This description has been generalized to a GLE-based model in two works by Hohenegger et al.^100,101^ Instead of applying a single-mode Maxwell model for the stress tensor (which would result in the Oldroyd-B model), they assumed that the memory can be expressed as a series of exponentials (see also section 6). In this way, they were able to describe, in very general terms, the movement of passive tracers in a viscoelastic medium.

## 6 Implementation of GLE Simulations and Efficient Integration

In the previous section, we have introduced and discussed various different models to incorporate non-Markovian dynamics into complex coarse-grained models. We have mostly skipped details of the numerical implementation and efficient integration of the equations of motion. These will be discussed in this section.

The first papers on the integration of stochastic differential equations based on the GLE date back to the 1980s. In a seminal contribution, Ermak and Buckholz proposed two novel approaches for the integration of a GLE in an arbitrary external potential.^102^ The first is based on a direct integration scheme that can be applied to arbitrary memory kernels, in which the memory integral is discretized in time using a standard midpoint rule and the noise is calculated using a convolution approach, similar to the Fourier transform method which will be introduced below.^123^ The second approach is based on the assumption that the memory kernel is exponential, which allows it to be replaced by an equivalent extended Markovian model with one additional variable. This method is based on an idea presented 1 year earlier by Ferrario and Grigolini,^124^ and it is the precursor of the auxiliary variable technique discussed below (see section 6.2). In the same year, Ciccotti et al. published two works^103,125^ in which they integrated the GLE by assuming a truncation of the continued fraction representation of the memory kernel,^126^ which is equivalent to the auto-regression model used by Marchesoni et al.^127^ and Smith et al.^95^

Generally, one faces two main issues when trying to integrate a GLE: first, the integration of the friction force which, in principle, requires the storage and evaluation of the entire past of all coarse-grained particles and, second, the generation of suitably correlated random numbers. In the most complex situation, where the system is governed by non-Markovian interactions between different particles, these random numbers must be correlated in *space and time*.^57,91^ Two distinct types of approaches have been used to solve these problems, the direct integration and the auxiliary variable methods. Both have their advantages and disadvantages, which we will discuss in the following.

### 6.1 Direct Integration

In the direct integration approach, the convolution integral appearing in the friction force is integrated numerically using a time cutoff *t*_cut_, which effectively corresponds to multiplying the memory kernel with a Heaviside theta function Θ(*t*_cut_ – *t*). This allows for a straightforward and easy evaluation; however, it can introduce artifacts. The most obvious artifact is that any long-time tails in the dynamics will be disregarded, which can be problematic in situations involving hydrodynamic tails (see the discussion in ref (57)). In most applications, however, in which the introduction of memory is supposed to be an improvement compared to the idealistic Markovian assumption, the cutoff is not expected to lead to serious errors.

One major challenge in direct integration methods is to produce suitably correlated random forces. The most popular approach is based on the original idea of Ermak et al.^102^ to express the colored noise as a convolution of an unknown function with a white noise variable. The method was successfully applied by Barrat et al.^123^ using a Fourier transform approach, but the function can also be determined by auto-regressive techniques^95^ or optimization.^29^ For non-interacting particles, the scaling of the method is similar to that of the direct integrator of the friction force; i.e., the computational costs increase linearly with the particle number *N* and the number of memory steps, *N*_*t*_ = *t*_cut_/*Δt* (where *Δt* is the time step) with the scaling .

Producing colored random numbers becomes much more problematic when simulating interacting particles or integrating multidimensional GLEs, in which the random force also has cross-correlations, described by the off-diagonal terms in the memory kernel matrix. This problem was addressed by Chen et al.^91^ and Jung et al.^57^ and, in both cases, was solved using the Lanczos method.^128^ In short, the Lanczos method can be used to approximate highly dimensional matrices by tridiagonal matrices in Krylov subspaces with significantly reduced dimension, thus allowing for efficient matrix inversion and Cholesky decomposition. If one can further assume that every coarse-grained dimension only interacts with a fixed number of “connected” variables (e.g., neighbors in particle-based descriptions), this method allows the computational time to be reduced to , making it suitable for applications in large-scale simulations.

The last remaining problem is the choice of an efficient GLE integrator. Generally, one can use any standard Langevin integrator, since the time-retarded contributions to the force can just be added to the total force on the coarse-grained variables. Addressing specifically GLEs, Tuckerman and Berne have derived a multiple time-stepping algorithm in 1991, which can be used in cases where the typical frequencies related to the conservative forces differ very much from the time scale of the memory.^97^ Jung et al.^56,57^ derived an alternative integrator which generalizes the Grønbech-Jensen/Farago Langevin (GJ-F) thermostat^129^ and was found to perform very well for both non-interacting and interacting particles.

The direct integration method is thus very flexible and can be applied to basically all non-Markovian models that were discussed in the literature. However, in cases where *N*_*t*_ is large, the computational overhead for the evaluation of the friction and the random force is significant.

### 6.2 Methods Based on Auxiliary Variables

The central idea of auxiliary variable approaches is to introduce additional stochastic variables and replace a GLE by an equivalent extended system of Markovian LEs. Let us consider a Markovian LE for two coupled degrees of system. As we will show below, integrating out one of them automatically results in the emergence of a memory kernel in the dynamical equation for the other.^22^ Inverting this procedure, one can transform a system with exponential memory into an *extended Markovian* system with an additional, auxiliary variable that mimics the effect of the memory. The auxiliary variable approaches use this fact to construct extended Markovian models for the GLE. The idea is to expand the memory kernel into multiple exponentials and then represent each one by an additional auxiliary variable.

Related approaches were already proposed in some of the very first works on numerical GLE integrators.^124,127^ In these studies, the auxiliary variables were constructed by a truncation of Mori’s continued fraction expansion.^126^ The method was revived about 10 years ago, mainly due to the work of Ceriotti et al., who used it as a practical numerical tool, in which the expansion is determined by a fitting procedure.^130−139^ Recently, two works have also extracted extended Markov models directly from fine-grained trajectories, with great success.^74,75^

To introduce the technique, let us consider the following two-dimensional linear differential equation^22^
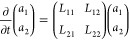
39

Solving eq 39 for the variable *a*_2_ and putting the result back in the equation for *a*_1_ gives
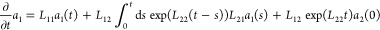
40

In eq 40, the dynamics of *a*_1_ now only depends on the initial conditions of *a*_2_ and not its time evolution. Instead, an integral term appears, which involves the history of *a*_1_. This procedure is exact and reversible. Since the Markovian eq 39 and the non-Markovian eq 40 are equivalent, it is evident that it should in many cases be possible to rewrite non-Markovian integro-differential equations such as GLEs in a Markovian form by the introduction of additional variables. Such a procedure allows one to describe the evolution of the convolution integral in a GLE in terms of a set of auxiliary variables, thus rendering the EoM Markovian.

This method was used by Ceriotti et al. to introduce a general framework for exploiting the GLE as a flexible thermostat in MD simulations.^130−135^ Following their scheme, a non-Markovian GLE of the form
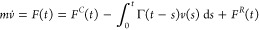
41can be rewritten in a Markovian form as
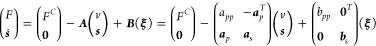
42where ***A*** and ***B*** are the drift and diffusion matrices, respectively. For canonical sampling, ***B*** is fully determined by ***A*** in terms of the FDT. The noise term **ξ** is a vector of uncorrelated Gaussian random numbers with zero mean and unity variance, which can be implemented rather efficiently compared to correlated noise. The vector ***s*** is a set of auxiliary variables, which effectively “stores” the dynamical history of *v*, while the drift matrix ***A*** includes the self- and cross-coupling of the momentum and the auxiliary variables. The matrix ***A*** must satisfy the requirement that ***A*** + ***A***^*T*^ is positive (semi)definite to ensure that ***B*** can be chosen in a manner consistent with the FDT and that a stationary distribution of (*v*, ***s***) exists. This can be ensured by choosing the nondiagonal elements in the drift matrix to be antisymmetric and the diagonal elements to be positive or zero, *A*_*ii*_ ≥ 0.

As long as this specific condition is met, one has some freedom in the choice of ***A***. For certain functional forms of the memory kernel Γ(*t*), equivalent parametrizations for eq 42 were proposed. Ceriotti et al. proposed parametrizations for exponential memory kernels and memory kernels that are δ-correlated in Fourier space.^131^ The total memory kernel can also be constructed as a sum of contributions, which allows, for example, one to use a sum of exponentials to describe memory decaying on different time scales^140^ or to approximate a power-law memory kernel.^135^ The δ-like memory kernel is defined by its amplitude, mean value, and a line width in Fourier space, which allows one to define a memory kernel with an arbitrary power spectrum by a sum of δ-like functions.^131^

As a side note, we remark that Ceriotti et al. did not have dynamic coarse-graining in mind in their work but rather the development of enhanced sampling schemes for MD simulations. Applying thermostats with memory and colored noise allows one to control and optimize the correlation times of modes with different frequencies independently.^131^ Ceriotti et al. also proposed to use nonequilibrium GLEs (with colored noise that does not fulfill the FDT) to mimic the effect of nuclear quantum fluctuations.^132^

Similar approaches can be used to parametrize memory kernels in GLE simulations.^29,67,74^ Li et al. considered a star polymer melt with the dynamics of a single-star polymer mapped onto a GLE and exploited the Volterra inversion method (eq 27) for the extraction of the memory kernel.^29^ In the CG simulations, they compared the results obtained using a discretized calculation of the convolution kernel with those using the auxiliary variable approach due to Ceriotti.^131^ Both methods were found to reproduce the VACF with small deviations on large time scales, which were, however, more pronounced in the discretized convolution integral approach. The direct calculation of the convolution integral necessarily involves cutting off the number of time steps considered in the evaluation of the GLE. In particular, if the memory kernel exhibits a slowly decaying tail, this will always lead to a overestimation of the dynamics due to the truncation of the long-time friction. The auxiliary variable approach shows a similar but slightly lower deviation. For the parametrization of the auxiliary variable approach, a set of damped oscillators was used. This fitting procedure allows one to represent the memory kernel for larger time scales, which can enhance the representability of the long-time-scale behavior. Even though the memory kernel is not truncated in the auxiliary variable approach, approximating long-tail memory kernels by a finite sum of exponentials still implicitly results in a, though less severe, truncation error.

The same approach was also applied to solutions ranging from generic star polymer solutions to a solution of tri-*n*-butyl phosphate in chloroform.^67^ In these systems, it was possible to capture the long-time scaling of the memory kernel accurately enough to match the VACF over all time scales with a reasonable number of fitting functions for the memory kernel. Furthermore, the authors established a GJ-F integrator^129^ for the auxiliary variable approach, thus enhancing the performance of the CG simulation due to larger time steps.

Li et al. also extended the auxiliary variable approach for the case of non-Markovian DPD equations and derived it in a pairwise-decomposed form, including also complex exponentials which allows for a better representation of the memory kernel.^29^ Here, the auxiliary variables were coupled to the relative velocities of the bead pairs, instead of the absolute velocity of a single particle. This leads to an increase in the computational cost compared to the GLE thermostat, as auxiliary variables must now be introduced for each bead pair. Nonetheless, it was found that this approach is roughly 20 times more efficient than the direct evaluation of the convolution integral in the same system.^98^ The authors demonstrated that both approaches can well capture the dynamical properties of the underlying FG system.

The auxiliary variable approach is thus clearly more efficient than the direct integration technique which we have discussed in section 6.1. One challenge is an accurate reconstruction of the non-Markovian dynamics, which often requires fitting of memory kernels with a series of (complex) exponentials. This problem, however, might not be very severe, because it is often not necessary to reproduce memory kernels in full detail. Furthermore, recent work on direct optimization has demonstrated that it is possible to faithfully represent self-friction memory kernels over several orders of magnitude in time with auxiliary variables.^74^

On the other hand, when looking at multidimensional memory kernels with distance-dependent pair memory contribution, the approach may also fail and it may not be possible to find an equivalent representation of the form of eq 42. The problem is that the different entries in the memory kernel matrix then depend on the relative distances between all particles in the system, and there is no (obvious) way to ensure that this memory kernel matrix is always positive (semi)-definite (see the discussion in the Appendix of ref (57)).

## 7 Physical Impact of Memory

From the point of view of dynamic coarse-graining, it is clear that memory effects should be included in CG models in many cases in order to *quantitatively* reproduce the dynamics of the underlying FG model. In addition, memory can have a significant impact on the *qualitative* behavior of materials. One particularly prominent example is the glass transition, which has been the subject of intense research for almost a century now and will not be discussed here (see refs (85, 87, and 141−145) for recent advances and reviews). Another important field where memory plays a central role is anomalous diffusion (see section 4.1), which has also attracted enormous interest due to its many applications in physics and biology and will also not be discussed here (for reviews, see, e.g., refs (59, 86, and 146)). There are many other cases where memory has a physical impact on systems, and we will now illustrate this using a few selected examples.

Mankin and co-workers studied the influence of memory on the motion of trapped Brownian particles in oscillatory viscoelastic shear flow with a power-law-type memory kernel.^147,148^ Among other things, they discovered a dynamic phase transition from a trapped to a diffusive state when increasing the memory exponent. Moreover, the cross-correlation of the particle motion in flow and shear direction changed sign twice with increasing exponent.

Lesnicki et al.^61^ gave a beautiful example of how the analysis of memory kernels can enhance the understanding of physical phenomena. They performed an accurate calculation of the memory kernel of a tagged LJ particle in a bath consisting of equivalent particles on long time scales, using the method of Carof et al.,^60^ and numerically derived the algebraic long-time tail for the memory kernel. They related this result to the Basset–Boussinesq hydrodynamic force equation, which is typically used to model colloidal spheres in suspension.^149,150^ Thus, they showed that the Basset–Boussinesq equation is also applicable in the microscopic regime, with parameters that can be directly derived from the memory function.^61^ Seyler and Presse^151,152^ investigated the influence of this “Basset history force” on the motion of microspheres in oscillatory flow and a periodic potential. They showed that hydrodynamic memory significantly enhances the mobility of microspheres and helps them to escape potential wells in which they would otherwise remain trapped for much longer times.

Goychuk^153,154^ considered the effect of hydrodynamic memory on the diffusion in so-called washboard potentials, where the diffusion is enhanced by orders of magnitude already in the absence of any memory.^155^ He showed that hydrodynamic forces can enhance the diffusion even further in such systems and induce a transient but long-lived superdiffusion regime, where the mean-square displacement scales with *t*^3^.

**Figure 4:**
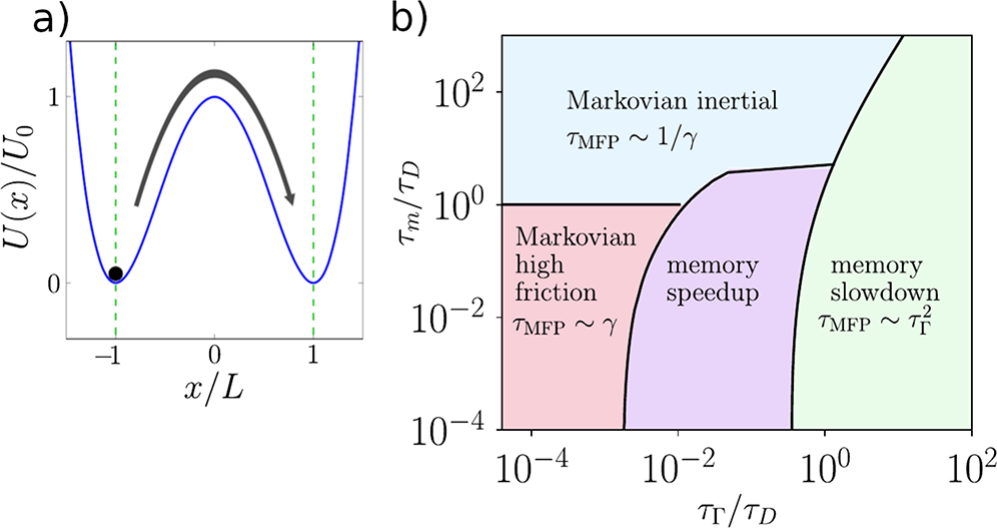
Effect of memory on the barrier crossing dynamics of a single particle.^47^ (a) Illustration of the simulation setup. (b) The important regimes for the mean first passage time, τ_MFP_: the Markovian regimes for overdamped and underdamped dynamics in which the memory has no effect, as well as the regimes in which the memory introduces a speedup or a slowdown compared to the Markovian results. τ_*m*_ and τ_*D*_, inertial and diffusive time scales; τ_Γ_, time scale of the memory kernel. Reprinted with permission from ref (47). Copyright 2018 AIP Publishing.

The above situations have in common that the memory kernels were long-range in time. However, memory effects may also qualitatively affect the dynamics of systems if the memory kernels are short-range, i.e., decay exponentially. One such example was recently discussed by Kappler et al.,^47^ who analyzed the influence of memory with an exponentially decaying memory kernel on the mean first passage time (MFPT), τ_MFP_, in a generic symmetric double well potential (see Figure 4). For fixed inertial and diffusive time scales, τ_*m*_ and τ_*D*_, they reported an intriguing non-monotonous behavior as a function of the time scale τ_Γ_ of the memory kernel, where the MFPT first decreases with τ_Γ_ (“memory speedup” regime in Figure 4) and then grows as τ_Γ_^2^ for large τ_Γ_ (“memory slowdown” regime in Figure 4). If multiple memory time scales τ_Γ,*i*_ with different associated friction constants γ_*i*_ are involved, then the behavior of the MFPT is dominated by the time scale τ_Γ,*j*_ for which γ_*j*_/τ_Γ,*j*_^2^ is the largest.^140,156^ This study demonstrated that, remarkably, memory effects in the presence of conservative interactions can affect the long-time dynamics far beyond the time scale of the memory. It further showed that this effect strongly depends on the chosen barrier height.

The findings of Kappler and co-workers might provide a possible explanation for the observation that Markovian DPD models can capture the long-time dynamics rather well in simple liquids with low viscosity, in which energy barriers are significantly smaller than *k*_B_*T*, whereas they tend to overestimate the diffusion coefficient in systems in which energy barriers due to conservative interactions are rather high—as is the case for polymer melts and solutes in polymer networks.^37^ In both cases, the separation of time scales is incomplete. However, the diffusion of the polymer should not depend much on the local relaxation processes in dilute polymer solutions and thus can be well captured by a Markovian approximation after appropriate time-scale mapping.^37^ In the case of penetrant diffusion in a polymer matrix, which is closely related to the MFPT problem in a double-well potential, one observes significant deviations from Markovian DPD models. A potential enhancement of the barrier crossing rate resulting from the Markovian approximation can accumulate over time and effectively translate into an enhanced diffusion coefficient.^37^

Memory effects can also be prominent in driven and active systems. Russo et al.^114^ derived a generalized dynamic density functional framework for reactive multicomponent fluids with memory and showed that reaction-diffusion equations for components with dissimilar memory kernels exhibit novel Turing patterns. Two examples of memory effects in systems of microswimmers were recently discovered by Nagai and co-workers^157^ and by Narinder and co-workers.^158^ Nagai et al.^157^ investigated the effect of memory (colored noise) on the pattern formation in fluids of microswimmers and showed that memory can induce a whole spectrum of novel patterns in such systems, including vortex lattices and laning. Narinder et al.^158^ studied the motion of colloidal microswimmers in a viscoelastic fluid both experimentally and theoretically and showed that memory can induce spontaneous circular motion.

These examples show how memory can fundamentally influence the dynamical behavior of systems. In many cases, properly accounting for memory effects in coarse-grained simulations is not just necessary to establish a proper quantitative link between the fine-grained and coarse-grained systems. It may also be crucial to capture the essential characteristics of the dynamics at the coarse-grained level.

## 8 Outlook

Over the past decade, a lot of progress has been made toward improving dynamical consistency in CG simulations based on the Mori–Zwanzig theory. While the Markovian approach has been exploited with varying success, in most cases, the systems under study were chosen such that the approximation is evidently valid. In such cases, even though the methodology could be validated, its applicability to real physical systems remains questionable. In general, for a moderate level of coarse-graining at a high density, the Markovian approximation is not valid. Interestingly, the approximation could still capture the long-time dynamics of simple liquids where the time scales are not well separated.^37^ For multibead mapping schemes in polymer systems at high densities, the approximation introduces errors in the long-time dynamics, probably due to the comparable time scales of memory effects and chain relaxation processes that govern diffusion. In principle, this could be circumvented by choosing a higher degree of coarse-graining, which would enhance the time-scale separation. However, such models will ultimately lose their predictive capabilities, as the mapping scheme for a given physical question is chosen based on the corresponding length and time scales of interest.

On the other hand, non-Markovian CG models are more flexible and can be applied to a broad range of physical problems, with an obvious increase in computational overhead. Among the existing methods, the generalized Langevin dynamics method^57^ proposed by Jung et al. is rather general and can be applied to any physical system with arbitrary mapping schemes, however, at a relatively high computational cost. The assumption of pairwise-additivity of the frictional forces as proposed by Li et al. allows one to formulate non-Markovian DPD-type models, which can be integrated more efficiently using auxiliary variable approaches.^29^ While this circumvents the computational overhead of explicit memory evaluation to a large extent, the non-Markovian DPD models with a moderate degree of coarse-graining can still be less efficient compared to fine-grained MD simulations, again limiting their applicability to coarser models.

Unfortunately, there are no studies yet in which the predictive capabilities of non-Markovian DPD models are demonstrated conclusively. If the corresponding memory kernels (which only depend on the direct interactions and, thus, local correlations) can be assumed to be short-lived compared to the diffusive time scales and the dynamics on longer time scales are partially encoded in the conservative interactions, it is reasonable to assume that non-Markovian DPD models can be parametrized with relatively short fine-grained MD simulations, while the dynamics on long time scales can be sampled with the CG models. One possible application of this kind would be the penetrant diffusion in polymer melts or polymer networks, for which it was shown that Markovian DPD approaches do not correctly reproduce diffusion.^37^ However, to the best of our knowledge, no study has applied any of the discussed non-Markovian CG approaches to predict dynamical properties of such materials or related molecular processes. One possible reason is the nontrivial derivation of the memory kernels and the rather complicated and computationally expensive implementation of the CG model.

Transferability of CG models is another important issue that requires future attention, in particular with respect to dynamical properties. In the field of systematic polymer coarse-graining, transferable pair potentials have been developed based on approaches that minimize the contributions of average, and strongly state-dependent, multibody effects. The CRW pair potential^45,46^ and the EF-CG pair potential^48^ represent the free energy associated with the interactions among the internal DoF of two beads at a fixed distance, excluding contributions of the nonbonded environment of the two beads. CRW models for linear alkanes are shown to be transferable between the melting and boiling points of the materials,^46^ reproduce the liquid surface tension, have been used to study wetting problems,^159^ and, applied to syndiotactic polystyrene,^160^ have been successfully used to study crystallization in the bulk^161^ and at the surface of a thin polymer film.^162^ These studies rely on the transferability of the potential and have been applied to static aspects of problems whose dynamics is of significant interest too. The Markovian MZ-DPD approach has, with an eye to transferability, been derived based on EF-CG interactions, while neglecting (state-dependent) multibody contributions to the DPD pair frictions.^37^ This approach, in principle, requires time-scale separation, i.e., distances between beads are fixed on the time scale of the memory kernel, and is expected to work for polymer-based systems such as polystyrene in which rotations of side groups occur on time scales where the monomeric units hardly move. This system is also a good example for testing the temperature transferability of memory kernels employed in a non-Markovian extension of the work in ref (37), e.g., with respect to reproducing temperature-dependent segmental and chain dynamics of polystyrene.^163^

While the parametrization of the original DPD model, which is often applied to simple bead–spring polymer systems, is generic (not chemistry specific), it can still capture some fundamental dynamical properties of well-known theoretical models in polymer physics, even though it fails to capture reptation dynamics for long polymer chains in melts.^164^ In this line, it is conceivable that an in-depth understanding of friction and memory kernels and its coupling to the conservative interactions can be utilized to establish a similar top-down procedure to derive CG models with realistic dynamics. The realm of non-Markovian simulations, in principle, allows one to tune the dynamical properties of generic CG models with a greater flexibility, opening new possibilities in the development of empirical models with a broader range of possible applications.

Beyond the realm of equilibrium systems, the MZ theory and the application of GLEs has been extended to nonequilibrium and nonstationary processes.^54,55^ For example, non-Markovian dynamics emerges naturally when looking at “hot Brownian motion”, i.e., the motion of heated colloids in a fluctuating thermodynamic environment.^165,166^ Non-Markovian interactions with time delay offer interesting opportunities for a feedback control of Brownian motion and create intriguing novel equilibrium states.^167,168^ These examples illustrate that a modification of dissipative and stochastic interactions in nonequilibrium can have a qualitative impact on the *structural* properties of the system (see, e.g., ref (157)). One problem along this line will be that, in nonequilibrium, a clear distinction between systematic and random forces is missing,^169,170^ which makes it challenging to establish a meaningful, systematic dynamic coarse-graining procedure.

In the following, an (incomplete) list of open questions and problems is given, that could potentially guide future research toward practical applications of non-Markovian models.

- Understanding the transferability and predictive power of (equilibrium) non-Markovian models.
- Implementation of (distance-dependent) pairwise friction kernels could be essential to achieve a high level of transferability. Potential issues of currently proposed (particle-based) techniques that should be addressed are
  - the assumption of a time-scale separation between the decay of the memory kernel and the characteristic diffusion time of the particles,
  - the usage of auxiliary variable approaches for models with self- and pair-memory kernels,
  - and the handling of long-range and long-time interactions.
- The practical application of the coarse-graining techniques in nonstationary and nonequilibrium systems. This will include
  - analysis of the FDT for nonequilibrium processes and in nonstationary situations,
  - the development of practical computational tools for the time-integration of nonequilibrium coarse-grained models,
  - and further development of reconstruction techniques for nonstationary memory kernels.
- The application of state-of-the-art techniques to the problem of non-Markovian coarse-graining. This mainly includes the usage of machine-learning tools,^74^ which have the potential to be a powerful methodology to approach some of the above listed open problems.

A multidisciplinary, collaborative effort will be needed to standardize the methodologies and exploit their potential while reaching a broader community of researchers. Concrete application to relevant physical questions would help drive continuous improvements on the methodological front and broaden their capabilities.

## List of Acronyms

ACF: Auto-correlation function
CG: Coarse-grained
CRW: Conditional reversible work
DoF: Degrees of freedom
DPD: Dissipative particle dynamics
EF-CG: Effective force-coarse-graining
EoM: Equation of motion
FACF: Force auto-correlation function
FDT: Fluctuation–dissipation theorem
FG: Fine-grained
GJ/F: Grønbech-Jensen/Farago integrator
GLE: Generalized Langevin equation
IBI: Iterative Boltzmann inversion
IMR: Iterative memory reconstruction
IMRF: IMR based on FACV
IMRV: IMR based on VACF
ISF: Intermediate scattering function
LE: Langevin equation
LJ: Lennard-Jones
MD: Molecular dynamics
MFPT: Mean first passage time
MSD: Mean-square displacement
MZ: Mori–Zwanzig
NM-DDFT: Non-Markovian dynamic density functional theory
RDF: Radial distribution function
VACF: Velocity auto-correlation function
